# Single-cell RNA sequencing of human non-hematopoietic bone marrow cells reveals a unique set of inter-species conserved biomarkers for native mesenchymal stromal cells

**DOI:** 10.1186/s13287-023-03437-x

**Published:** 2023-08-30

**Authors:** Loïc Fiévet, Nicolas Espagnolle, Daniela Gerovska, David Bernard, Charlotte Syrykh, Camille Laurent, Pierre Layrolle, Julien De Lima, Arthur Justo, Nicolas Reina, Louis Casteilla, Marcos J. Araúzo-Bravo, Abderrahim Naji, Jean-Christophe Pagès, Frédéric Deschaseaux

**Affiliations:** 1 RESTORE, Université de Toulouse, EFS Occitanie, INP-ENVT, Inserm U1301, UMR CNRS 5070, France, Université de Toulouse, Toulouse, France; 2 CHU de Toulouse, IFB, Hôpital Purpan, Toulouse, France; 3 Group of Computational Biology and Systems Biomedicine, Biodonostia Health Research Institute, 20014 San Sebastián, Spain; 4 Basque Foundation for Science, IKERBASQUE, 48009 Bilbao, Spain; 5 Department of Cell Biology and Histology, Faculty of Medicine and Nursing, University of Basque Country (UPV/EHU), 48940 Leioa, Spain; 6 Department d’Anatomie Pathologique, Institut Universitaire du Cancer, CHU de Toulouse, Toulouse, France; 7 Tonic Inserm/UPS UMR 1214, CHU Purpan Hospital, Toulouse, France; 8 UMR 1238 Inserm, Phy-OS, Bone Sarcoma and Remodeling of Calcified Tissues, School of Medicine, University of Nantes, Nantes, France; 9 Department de Chirurgie Orthopédique, Pierre Paul Riquet, Hôpital Purpan, Toulouse, France; 10 Department of Environmental Medicine, Cooperative Medicine Unit, Research and Education Faculty, Medicine Science Cluster, Nankoku, Kochi Prefecture Japan

**Keywords:** Mesenchymal stromal cells, Single-cell RNA sequencing, Characterization, Transcriptomic, Human bone marrow, Biomarkers

## Abstract

**Background:**

Native bone marrow (BM) mesenchymal stem/stromal cells (BM-MSCs) participate in generating and shaping the skeleton and BM throughout the lifespan. Moreover, BM-MSCs regulate hematopoiesis by contributing to the hematopoietic stem cell niche in providing critical cytokines, chemokines and extracellular matrix components. However, BM-MSCs contain a heterogeneous cell population that remains ill-defined. Although studies on the taxonomy of native BM-MSCs in mice have just started to emerge, the taxonomy of native human BM-MSCs remains unelucidated.

**Methods:**

By using single-cell RNA sequencing (scRNA-seq), we aimed to define a proper taxonomy for native human BM non-hematopoietic subsets including endothelial cells (ECs) and mural cells (MCs) but with a focal point on MSCs. To this end, transcriptomic scRNA-seq data were generated from 5 distinct BM donors and were analyzed together with other transcriptomic data and with computational biology analyses at different levels to identify, characterize and classify distinct native cell subsets with relevant biomarkers.

**Results:**

We could ascribe novel specific biomarkers to ECs, MCs and MSCs. Unlike ECs and MCs, MSCs exhibited an adipogenic transcriptomic pattern while co-expressing genes related to hematopoiesis support and multilineage commitment potential. Furthermore, by a comparative analysis of scRNA-seq of BM cells from humans and mice, we identified core genes conserved in both species. Notably, we identified *MARCKS*, *CXCL12*, *PDGFRA*, and *LEPR* together with adipogenic factors as archetypal biomarkers of native MSCs within BM. In addition, our data suggest some complex gene nodes regulating critical biological functions of native BM-MSCs together with a preferential commitment toward an adipocyte lineage.

**Conclusions:**

Overall, our taxonomy for native BM non-hematopoietic compartment provides an explicit depiction of gene expression in human ECs, MCs and MSCs at single-cell resolution. This analysis helps enhance our understanding of the phenotype and the complexity of biological functions of native human BM-MSCs.

**Supplementary Information:**

The online version contains supplementary material available at 10.1186/s13287-023-03437-x.

## Background

In adults, bone marrow (BM) is a soft tissue restricted to bones that supports the production of blood cells, including immune cells. The current model of hematopoiesis is hierarchical, with hematopoietic stem cells (HSCs) at the origin of all hematopoietic cells. HSCs reside in a defined microenvironment, the BM niche [1], consisting of a specialized matrix and a set of cells expressing biological signals preventing their maturation and stimulating proliferation while preserving stemness [2]. In addition to hematopoietic cells, BM contains a set of non-hematopoietic cells belonging to osteo-chondroblastic and adipocyte lineages. Besides containing bone cells, the BM medullar cavity is richly vascularized. Therefore, vessels are an important compartment of the BM made of endothelial cells (ECs) and mural cells (MCs) including pericytes and vascular smooth muscle cells. Remarkably, in the HSC niche, bone-forming cells and perivascular cells can be generated from BM mesenchymal stromal cells (MSCs). Transplantation experiments revealed that HSCs are located in perivascular and endosteal sites in close contact with MSCs, which suggests functional relations between these two types of cells [3–5].

MSCs were described by Friedenstein et al. [6] for their ability to generate colony-forming unit-fibroblasts (CFU-Fs) and to sustain hematopoiesis in vitro [7] and in vivo [8]. MSCs are multipotent and can differentiate into osteoblasts, adipocytes, chondrocytes and vascular smooth muscle cells [9–11]. Despite numerous data describing MSCs, markers identifying MSCs in humans remain elusive. Because MSCs expand easily in vitro, most investigations were performed with cultured cells. This approach might affect the native identity of MSCs. Markers for native MSCs have been defined in mice (e.g., CXCL12, NG2 or chondroitin sulfate proteoglycan 4, NESTIN, leptin receptor [LEPR]) [12–18]. Studies of humans suggested CD90 (THY1) [19], CD105 (Endoglin), CD73 (NT5E; 5′-Nucleotidase Ecto) [20], CD146 (MCAM; Melanoma Cell Adhesion Molecule) [8], CD106 (VCAM1; Vascular Cell Adhesion Molecule 1) [21], CD271 (LNGFR; Low-Affinity Nerve Growth Factor Receptor) [22], CD140a (PDGFRA; Platelet Derived Growth Factor Receptor Alpha), CD200 (OX-2) [23] and CD49a [24] as native MSC markers. However, because of potential heterogeneity, the native phenotype of BM-MSCs needs to be confirmed at the single-cell level. In addition, some of these makers are shared by other BM cells such as hematopoietic cells, MCs (e.g., pericytes and vascular smooth muscle cells) or ECs [25].

The advent of single-cell sorting and RNA sequencing allows for a deep characterization of cells from living tissues. Regarding the heterogeneity and complexity of the HSC niche, single-cell RNA sequencing (scRNA-seq) offers to resolve the characterization of BM-MSCs in mice and humans [11, 26–30]. However, to avoid bias, scRNA-seq experiments must consider experimental issues such as the processing of samples from different individuals and sexes.

In the present work, we used scRNA-seq of male/female human BM samples and multimodal analyses comparing humans and mice to define a core of highly conserved MSC markers consisting of secreted proteins, transcription factors (TFs) and regulators of translation. This set highlights functions specific to BM-MSCs, such as extracellular matrix (ECM) production and hematopoietic or bone growth factors. Moreover, this analysis allowed us to propose a list of new factors at the center of molecular pathways controlling key functions in MSCs. We also reveal transcriptomes of two other non-hematopoietic BM cells (i.e., ECs and MCs).

## Methods

### Isolation of human BM-enriched stromal population

**Fig. 1:**
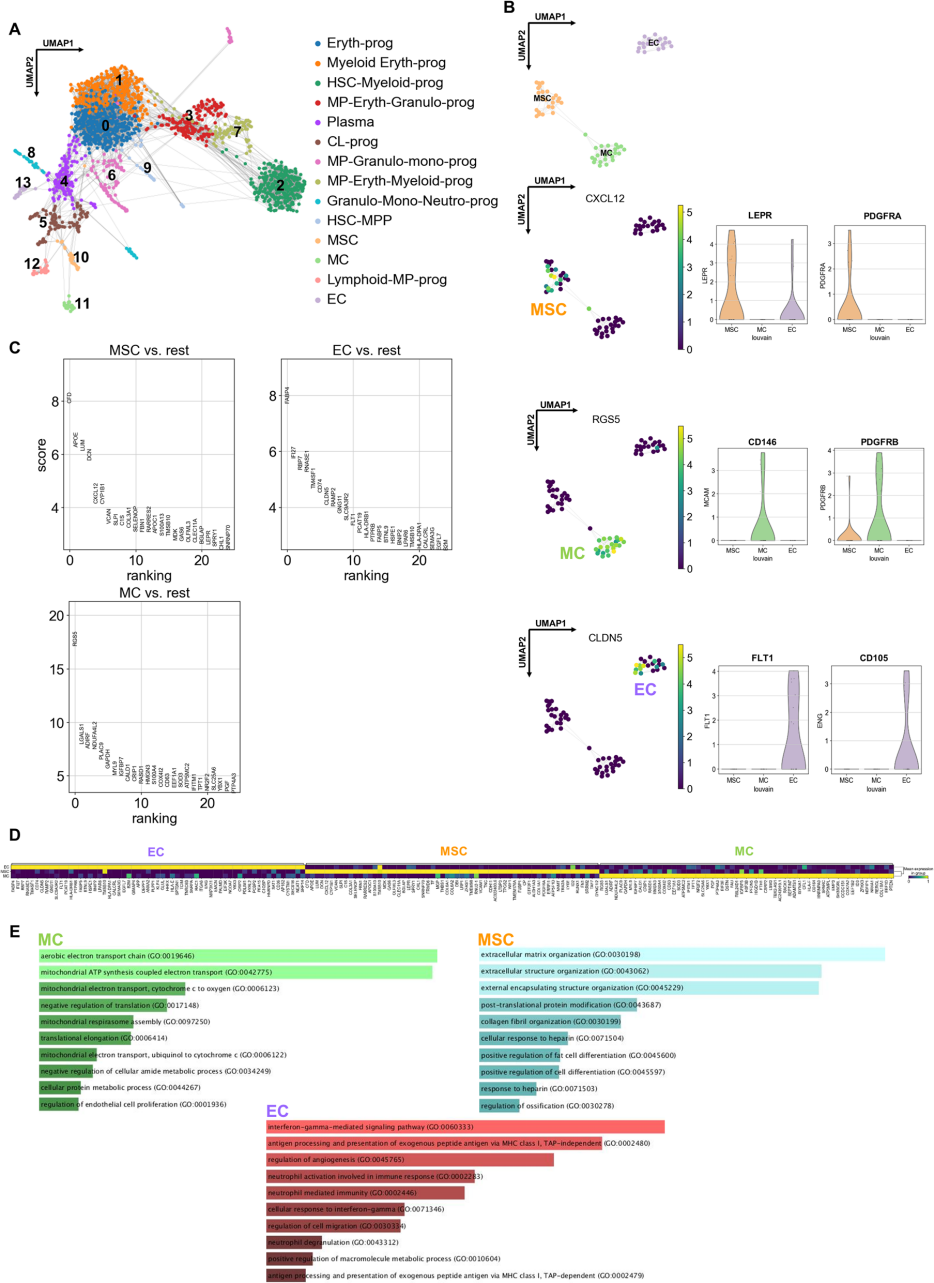
Human adult bone marrow (BM) single-cell sequencing analysis. **A** Fourteen CD45-/low, CD235- BM sorting cell clusters were obtained after analysis. UMAP visualization of 1677 cells (*n* = 5), annotated post hoc and colored by Louvain clustering. **B** Non-hematopoietic cluster identification and depiction by UMAP and violin visualizations, annotated post hoc with their most representative marker: *CXCL12*, *PDGFRA* and *LEPR* for mesenchymal stem/stromal cells (MSCs), *RGS5*, *CD146* and *PDGFRB* for mural cells (MCs), and claudin 5 (*CLDN5*), *FLT1* and *CD105* for endothelial cells (ECs). **C** Top genes expressed by MSCs (10), MCs (11) and ECs (12) after analysis of differentially expressed genes (DEGs). Data are depicted as genes significantly more expressed compared to the other clusters of cells. **D** Top 60 DEGs (columns) for each non-hematopoietic cell subsets (rows labeled on top of diagram) ranked after dendrogram analysis. **E** Gene Ontology (GO) terms of biological processes for DEGs for each non-hematopoietic cluster. Analysis calculated from Enrichr and ranked by combined score [75]

To isolate stromal cells from BM, fresh femoral heads were manually scraped. The bone fragments were incubated and shaken in collagenase NB4 (Serva Electrophoresis GmbH) and dispase (Roche) in αMEM + glutamax (Gibco) for 2 h at 37 °C. The bones were washed extensively with PBS (phosphate buffered saline; Gibco), and dissociated cells were collected. The mononuclear cell fraction was separated by density gradient centrifugation by using Products (Corp.) and counted. BM cells were frozen at − 80 °C in DMSO (dimethylsulfoxide) and thawed before sorting. More precisely, BM treated by droplet-based scRNA-seq included 3 men and 2 women (Fig. 1a + Additional file 1: Fig. S1) older than 60 years (mean ± SD = 70 ± 7 years; range 63–77).

Considering previous data, including ours, showing that human native BM-MSCs could be derived from the CD45-/low population of cells, we adopted to deplete cells strongly positive for CD45 by gating on the CD45-/low fraction as described [31, 32]. After CD45-positive cell depletion, live mononuclear cells from all samples underwent single-cell isolation to generate mRNA libraries.

### Flow cytometry

Cells were thawed and blocked in 0.1% human serum albumin (LFB Biomedicaments; Courtaboeuf, France) and then stained with CD45, GYPA, CD200, CD271, LEPR, CD49A (BD Biosciences; Franklin Lakes, NJ, USA); staining was analyzed with an ADP Cyan flow cytometer (Beckman Coulter; Brea, CA, USA). Cells were sorted by using BD LSRFortessa. Data were analyzed with Kaluza v1.2 (Beckman Coulter). Dead cells were excluded by DAPI staining (Sigma).

### Single-cell library preparation and sequencing

For scRNA-seq libraries, we sorted the CD45- GYPA- BM cellular subsets from 5 patients (TF166-256-261-263-273) on a BD FACS Aria Fusion cell sorter into 384-well plates. The libraries were generated by using the Chromium Controller Single-Cell Instrument and Chromium Single Cell 3′ Library & Gel Bead Kit v2 A Chip Kit and i7 Multiplex Kit (10×Genomics) according to the manufacturer’s protocol (manual part no. CG00052 Rev A). Libraries were run on an Illumina HiSeq4000-TORNADE as 150-bp paired-end reads, at one full lane per sample. Sequencing results were demultiplexed and converted to a FASTQ format by using Illumina bcl2fastq software. Alevin pipeline integrated with the salmon software (version salmon-0.14.1) was used to align to the GRCh38 transcriptome and build the (cell, UMI) expression matrix for each sample used [33]. To label the cellular components of the human BM stroma, we analyzed all human samples in the same microchip in order to minimize experimental procedure effects.

We obtained 14,947 cells expressing 60,179 genes that were used for downstream analysis using Scanpy [34]. Strong quality control filtered out 13,070 cells with less than 200 expressed genes (resulting a matrix of 1877 cells × 60,179 genes), and 45,003 genes detected in fewer than 3 cells (1877 cells × 15,178 genes). Next, we removed genes with gene names starting with "RP" (1877 cells × 14,903 genes). We also removed potentially dead cells expressing more than 7.5% mitochondrial genes and duplicate cells that express more than 3500 genes (1677 cells × 14,903 genes). We total-count normalized the data matrix to 10,000 reads to become comparable among cells and logarithmized the data. Finally, for the 1677 cells, we obtained a matrix with the 5009 most variable transcribed genes across these cells (min mean = 0.0125, max mean = 3, min dispersion = 0.5). We reduced the dimensionality of the data by running principal component analysis, which revealed the main axes of variation and de-noised the data for each batch. Subsequently, graph-based clustering was performed to group cells according to their gene expression profile (Fig. 1b). Uniform manifold approximation and projection (UMAP) plots were used for visualization.

### Visualization and clustering

**Fig. 2:**
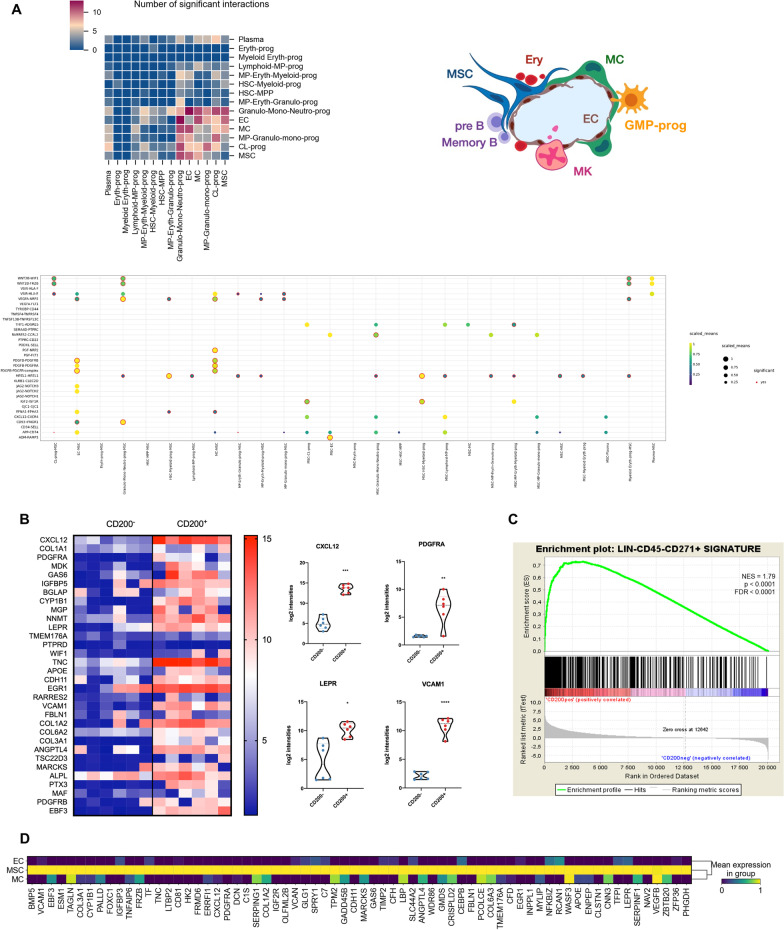
Human BM-MSC characterizations. **A** Representative CellphoneDB analysis matrix plot of ligand-receptor expression between each cluster and between MSCs, MCs, and ECs. The heatmap depicts the number of significant interactions between the different types of cells. Below, the interactions are defined by the type of receptor-ligand potentially active between MSCs and all of other clusters. **B** Heatmap representation of the genes expressed by native human BM-MSCs directly sorted (CD271 + /CD200 + /CD45−) and statistically compared to the CD271 + /CD200−/CD45− BM population. Study performed by Affymetrix mircoarrays. mRNA expression of representative markers of MSCs obtained from microarrays (*n* = 6; *p < 0.05; **p < 0.001; ***p < 0.0001). **C** Gene set enrichment analysis (GSEA) plot showing similarities between human BM CD271 + sorting cells from Li et al. study [76] and our study described above. Results were statistically significant according to normalized enrichment score (NES), false discovery rate (FDR) and *p* value depicted on the panel. **D** Distribution of the most correlated genes with the MSC subset from 193 enriched genes identified by GSEA analysis. Their expression was compared to that found for MCs and ECs

To visualize the data, we used the Scanpy package (https://scanpy.readthedocs.io/en/stable/index.html#) in Python language on a Jupyter Notebook with Anaconda interface software and further reduced the dimensionality of the entire 2825-cell dataset to project the cells in 2D space by using *t*-SNE or UMAP, on the basis of the aligned canonical correlation analysis. Aligned canonical correlation analysis was also used as a basis for partitioning the dataset into clusters using a shared nearest-neighbor modularity optimization algorithm. Using graph-based clustering, we divided the cells into 14 transcriptional subpopulations (Fig. 1). We then separated hematopoietic cells and non-hematopoietic cells to initially examine the transcriptional profile of microenvironment subpopulations (Fig. 2).

### Signature-based cell classification

To classify cells, we used a Louvain (or Leiden) algorithm implemented in Scanpy. Population-specific signatures were defined by the top 100 genes ranked by the significance analysis.

### CellPhoneDB analysis

We used the method 2 (see CellPhoneDB in Github) to model the different cell–cell interactions between the cell clusters we obtained and between MSCs and the rest in particular. This method retrieves interactions (which type of receptor interacts with their ligands) where the mean expression of the interacting partners (proteins participating in the interaction) displays significant cell state specificity by employing a random shuffling methodology.

### Pathway enrichment analysis

For all analyses with Enrichr, Omicsnet and Genomatix, associations were considered statistically significant at *q* < 0.05 (i.e., *p*‐values adjusted for multiple testing).

### Immuno-staining of human BM samples

Human BM biopsies were sliced and treated for immune-staining by using the following antibodies: anti-LEPR (goat anti-LEPR polyclonal Ab, PÄ5-18522; Invitrogen), anti MARCKS (polyclonal, PA5-84812, Invitrogen) and anti ACTA2 (clone 1A4, 14-9760-82, Invitrogen). Human biopsy tissue was fixed in 4% paraformaldehyde, embedded in paraffin, and sectioned at 6 μm. Slides were scanned (NanoZoomer; Hamamatsu, Photonics, Hamamatsu City, Shizuoka, Japan, http://www.hamamatsu.com) and observed by using the virtual microscope (NDP view; Hamamatsu). Histomorphometry of images was performed by using ImageJ software (National Institute of Health, Bethesda, MD). For immunofluorescence the Quadruple immunofluorescence (QIF) staining was performed using the BOND RX automated slide stainer (LEICA Biosystems) on 4 µm FFPE bone marrow slides. After baking and dewaxing, slides were heat pretreated using ER2 (pH9, LEICA Biosystems) pretreatment solution (40 min at 98 °C) and subsequently blocked for endogenous peroxidase using Discovery Inhibitor (ROCHE Diagnostics, 15 min RT). The QIF staining was performed in a 4-step protocol with sequential denaturation (ER1 buffer (pH6), 20 min at 100 °C, LEICA Biosystems) after each step. Antibody blocking preceded each primary antibody incubation step using the Antibody Diluent Block (AKOYA Biosciences) during 5 min at RT. Slides were incubated with primary anti-MARCKS (polyclonal, PA5-84812, Invitrogen, 1/200 in Envision Flex antibody diluent (Agilent Technologies)), anti-alpha smooth muscle actin (clone 1A4, 14-9760-82, Invitrogen, 1/100 in Envision Flex antibody diluent (Agilent Technologies)) and anti-CD31 (clone JC70, Ready to use, ROCHE Diagnostics) during 20 min at RT. The antibodies were detected using the Opal Polymer HRP (anti-mouse and anti-rabbit) detection system (10 min, RT, AKOYA Biosciences) before visualization using the Opal Polaris 570, Opal Polaris 650, Opal Polaris 520 and Opal Polaris 480 reagent packs subsequently (10 min at RT, AKOYA Biosciences). The tissue slides were counterstained using Spectral DAPI (10 min, RT, AKOYA Biosciences), and mounted with Prolong Gold Antifade reagent (Life Technologies). Fluorescent stained whole tissue slides were scanned in 16 bits using the Zeiss AxioScan.Z1 (Carl Zeiss, Oberkochen, Germany) whole-slide scanner equipped with a Colibri 7 solid-state light source and appropriate filter cubes.

### Statistical analysis

Statistical analysis—excluding that for RNA-seq experiments—involved using PRISM (GraphPad). Two groups were compared by unpaired *t*-test. Significance was defined at *p* < 0.05.

### Data availability

The data discussed in this publication have been deposited in NCBI's Gene Expression Omnibus (https://www.ncbi.nlm.nih.gov/geo/) and are accessible through GEO Series accession number GSE224152 for scRNA-seq and GSE198049 for microarrays.

## Results

### Characterization of non-hematopoietic cell subpopulations in human adult BM

After BM CD45^−/low^ cell selection and scRNA-seq processing, we identified 14 distinct cell subsets (Fig. 1A) according to their respective gene signatures. We further inferred their relationships and characterized diversities within specific cell types by using graph abstraction, correlations of average expression profiles between clusters (Additional file 1: Fig. S1), and diffusion map analysis [35]. Despite the CD45-/low selection, hematopoietic cells were still predominant among the 14 identified clusters (11/14). In fact, the resting hematopoietic fractions contained immature hematopoietic progenitors, HSCs or multipotent progenitors closely related to HSCs. Although cells from myeloid lineages formed the major fractions, we still detected multipotent lymphoid progenitors and some plasma cells and B-cell types (Additional file 1: Figs. S1 and S2) [36, 37].

We identified three fractions of genuine non-hematopoietic cells expressing markers of MSCs, MCs and ECs (Fig. 1B). *PDGFRA* + /*LEPR* + MSC clusters consisted of cells strongly expressing the HSC niche marker *CXCL12* (cluster 10), whereas the *FLT1* + /*ENG* + EC population (cluster 11) was annotated with the expression of *CLAUDIN 5* (*CLDN5*) and *PDGFRB* + /*MCAM* + MCs (cluster 13) with the pericyte marker regulator of G protein signaling 5 (*RGS5*) (Fig. 1B). We established a list of differentially expressed genes (DEGs) to phenotypically define these populations (Fig. 1C; Additional file 2: Table S1).

However, this classification was based on a scRNA-seq technology that is relatively limited to provide the whole transcriptome profile of these populations. We strengthened our data by comparison to published scRNA-seq data obtained from human BM-MSCs [38] (Fig. 2B–D) or human thymus ECs [39] and data from *Tabula sapiens* [40] scRNA-seq, which allowed for refining the annotation for all clusters obtained. Our results fitted well with these new annotations, thus confirming the correctness of our classification of MSCs (cluster 10) and ECs (cluster 13) (Additional file 1: Fig. S3). For instance, by using the *T. sapiens* data as a reference, ECs were found to match all EC populations (i.e., ECs, capillary ECs, ECs of arteries and the vascular tree). We annotated MCs (cluster 11) using the same procedure and showed that MCs had a shared profile with smooth muscle cells, vascular smooth muscle cells, pericytes and myofibroblasts. Finally, our MSC annotation was in accordance with the MSC fractions and other MSC-like cells such as fibroblasts and the stromal cell populations (Additional file 1: Fig. S3).

Regarding the gene expression and DEG results, ECs expressed the *CLDN5* endothelial tight junction molecule; FMS-related receptor tyrosine kinase 1 (FLT1), also known as vascular endothelial growth factor receptor 1 (*VEGFR-1*) receptor activity modifying protein 2 (*RAMP2*); and the fatty acid binding protein 4* (FABP4),* both known to be vascular markers (Fig. 1D). Other expressed genes were less characteristic but were known to be expressed by ECs: *IFI27*, *NHERF2*, *TM4SF1* or *GNG11*. The expression of some genes could be restricted to subtypes; for instance, retinol binding protein (*RBP7*) was detected in ECs with anti-oxidant activities [41] or transmembrane 4 L six family member 1 (*TM4SF1*) was reported as a key molecule for ECs during normal and tumoral angiogenesis [42]. Additionally to the gene expression, we sought to infer related functions with biological processes obtained by Gene Ontology (GO) analysis. For cells classified as ECs, the GO analysis indicated a vascular signature with regulation of perivascular cell differentiation (Fig. 1E, Additional file 3: TableS2), which reinforced the relevance of our analysis. These data also suggested that BM ECs could respond to immune challenge, with interferon gamma (IFNγ), receptors of immune cell markers, and genes expressing antigen receptors and antigen-processing molecules. In addition, GO terms of defense response were significant, a feature fitting well with the barrier function of ECs.

For MCs, our scRNA-seq analysis identified the *RGS5* (Fig. 1B, D); a well-known pericyte marker, *CD146,* also known as melanoma cell adhesion molecule (*MCAM*); platelet derived growth factor receptor beta (*PDGFRB*); myosin light and heavy chain 9 (*MYL9* and *MYH9*, respectively); caldesmon 1 (*CALD1*); and *SM22a* (transgelin [*TAGLN*]), all characteristic of perivascular smooth muscle cell-like pericytes (Additional file 2: Table S1). *ENDOSTATIN* (*COL18A1*) expressed by cells in MCs was shown to interact with EC function by inhibiting their proliferation [43]. Finally, this panel of genes agreed with the biological processes of MCs found on GO analysis (Additional file 4: Table S3) such as the regulation of EC proliferation and differentiation (GO:0001936), an expected function of MCs toward ECs. In addition, MC markers were significantly associated with strong metabolic activities, notably through the oxidative phosphorylation enabling production of adenosine triphosphate (Fig. 1E; Additional file 4: Table S3). Of note, comparison with cell-Atlas data from a public repository classified the MC fraction as pericytes with a mesenchymal origin of vascular smooth muscle lineage (Additional file 1: Fig. S3, Additional file 4: Table S3), which confirmed our annotation.

MSCs were positive for *CXCL12* hematopoietic niche factor and *PDGFRA*, a positive marker connected to human fetal BM CFU-F–initiating cells [44] and mouse native MSCs, independent of tissue origin [10]. *LEPR* expression in this subset of BM cells was previously described as a mouse pan-native MSC marker [16]. In addition, we observed numerous mRNAs coding for ECM components such as matrix Gla protein (*MGP*), versican (*VCAN*), collagen 3a1 (*COL3A1*), fibrillin1 (*FBN1*), and lumican (*LUM*) (Fig. 1C, D, Additional file 2: Table S1). Among the markers characterizing BM-MSCs, we identified new genes, myristoylated alanine rich protein kinase C substrate (*MARCKS*) and nicotinamide N-methyltransferase (*NNMT*) or zinc finger protein 36 (ZFP36). By focusing on MSC biological process, GO analysis (Additional file 5: Table S4) revealed genes of ECM organization (GO:0030198, GO:0043062), notably collagen (GO:0030199) and glycosaminoglycan (GO:0071504; Fig. 1E). Additionally, other annotated genes were involved in skeletal development, ossification (GO:0030278), tissue morphogenesis, and fat cell differentiation (GO:0045600). This finding was not surprising because multipotential BM-MSCs are at the origin of bones and other mesenchymal-derived tissues. In addition, using a scRNA-seq dataset from human CD45-/CD271 + BM-MSCs [45], we found that most genes characterized here were clustered with the MSC fraction (Additional file 1: Fig. S4), which strengthened our classification.

Overall, we identified three populations of non-hematopoietic cells from adult human BM: MCs, ECs and MSCs, these cells being at the origin of bone (osteo-chondroblastic cells and adipocytes) as well as the HSC niche. Additionally, BM ECs and MCs contributed to the vascular tree.

### Identification of molecular pathways regulating functions of human native BM-MSCs, ECs and MCs

Besides the determination of the MSC mRNA profile, we sought to determine specific molecular pathways in these non-hematopoietic BM cells. This analysis could help in understanding more precisely their functions in human adult BM. For this, we used repository bio-informatic tools with regularly curated data (Genomatix and Omicsnet). Among signaling pathways activated in MSCs, we noted, transforming growth factor β, PDGF, platelet-derived growth factor and SMAD pathways, known to be active in MSCs during osteogenesis, bone remodeling and vascular homeostasis regulation. The adipogenic pathway was also effective with the expression of *PPARγ*, *ADIPOQ*, *CFD*, *APOE* or *LEPR* (Additional file 1: Figs. S5, S6; Additional file 6: Table S5). The hematopoietic support was highlighted by numerous molecules found to be related with it (Additional file 1: Fig. S7) as well as the inflammatory response with the expression of *C7*, the delta sleep-inducing peptide immunoreactor *TSC22D3* and interferon-induced 35 kDa protein (*IFI35*) for example.

Next, we focused on TFs and mRNA binding proteins (mRNA BPs) expressed in BM-MSCs and found cAMP responsive element binding protein 1 (*CREB1*), early growth response 1 (*EGR1*), runt-related transcription factor 1 (*RUNX1*), *RUNX2*, sex-determining region Y-box 9 (*SOX9*), fibronectin 1 (*FN1*) and *ZFP36* (Additional file 1: Fig. S8). Most of these factors are known to regulate osteogenesis, adipogenesis and hematopoiesis (*RUNX1*, *RUNX2*, *SOX9*, *EGR1*, *FN1* and *CREB1*) [46–50]. The mRNA BP *ZFP36* is involved in inflammation related to tumor necrosis factor α and hypoxia [51].

For ECs, we observed angiogenic pathway signaling, notably through VEGF, cadherin, and HGF as key factors (Additional file 1: Fig. S9). TFs associated with ECs included signal transducer and activator of transcription 3 (*STAT3*), *ETS1*, *ETS2*, Krüppel-like factor 4 (*KLF4*), and *KLF2*, with known functions in endothelium [52–54]. *TCF7L2* activation was described only in corneal endothelium [55]. TFs or DNA/RNA BPs found in MC signaling pathways are involved in cell–cell or cell–ECM adhesion processes and regulation of angiogenesis (Additional file 1: Fig. S10), which are expected functions for this cell type. Y box binding protein 1 (*YBX1*), nuclear receptor subfamily 4 group A member 1 (*NR4A1*) and *NR2F2* were not reported to be associated to MCs, so they could represent new markers.

Finally, we studied interactions between MSCs and BM cells, ECs and MCs via the expression of ligand-receptors by using CellphoneDB analysis (Fig. 2A). This showed the preferred interactions between MSCs and ECs and MCs but also with some myeloid progenitors (Granulo-Mono-Neutro and MP-Granulo-mono progenitors). Furthermore, the main cytokine/growth factors linking MSCs, MCs and ECs were PDGF/PDGFR complexes, VEGF/VEGFR (NRP2, Neuropillin-2), placental growth factor (PGF), and insulin-like growth factor (IGF) pathways. Non-hematopoietic cells were also found to interact with hematopoietic stem/progenitor cells by different means encompassing cytokines to their receptors (WNT2B-FRZB, IGF2-IGF1R or CXCL12-CXCR4 as examples), membrane adhesion molecules such as CD34-SELL (Selectin L) or other types of interactions including the NOTCH pathway (JAG2-NOTCH1,2 or 3).

### Revealing a conserved phenotype and functions of human native BM-MSCs

With a comparison of the present gene lists with those from *T. sapiens* [40] and Ye et al. [45], we envisioned a marker-based phenotype for human BM-MSCs. Although scRNA-seq is a reliable technology for characterizing any cell type, results may vary according to numerous factors such as sample harvesting, cell sorting, RNA sequencing and analyzing. Therefore, we sought a minimal phenotype of MSCs that was conserved independent of the cell sources and cell selection protocols.

We took advantage of data we obtained by microarrays to directly phenotype selected BM-MSCs (GSE198049 from the GEO database). With the combination of two native BM-MSC markers CD271 [22] and CD200 [10, 56], we found that these CD271 + /CD200 + MSCs expressed a significantly increased list of markers with strong expression of *CXCL12*, *PDGFRA, LEPR* and *VCAM1* (Fig. 2B), in agreement with scRNA-seq data. In addition, for deeper analysis, we included gene arrays from another study [57]. We retrieved a list of genes that were commonly expressed by these directly selected BM-MSCs by performing gene set enrichment analysis (GSEA) (Fig. 2C). When intersected with our scRNA-seq data, we observed a clear enrichment in genes forming a MSC cluster not shared by ECs or MCs (Fig. 2D; Additional file 7: Table S6). By scrutinizing these common genes, we noted several key elements. In addition to the above MSC markers, we observed *EBF3*, a crucial TF in mouse MSCs [58], and *CDH11* and *ALPL*, which were previously detected in native MSCs [59]. For this MSC-conserved minimal gene list, we then used GO analysis to identify key biological functions. Meaningful terms were binding of growth factors (notably PDGF and IGF); formation of ECM constituents, including glycosaminoglycans (a panel of collagens, versican, laminin, decorin, tenascin, etc.); integrin binding; and peptidase regulator (Additional file 8: Table S7). All these GO terms related to expected activities for MSCs.

**Fig. 3:**
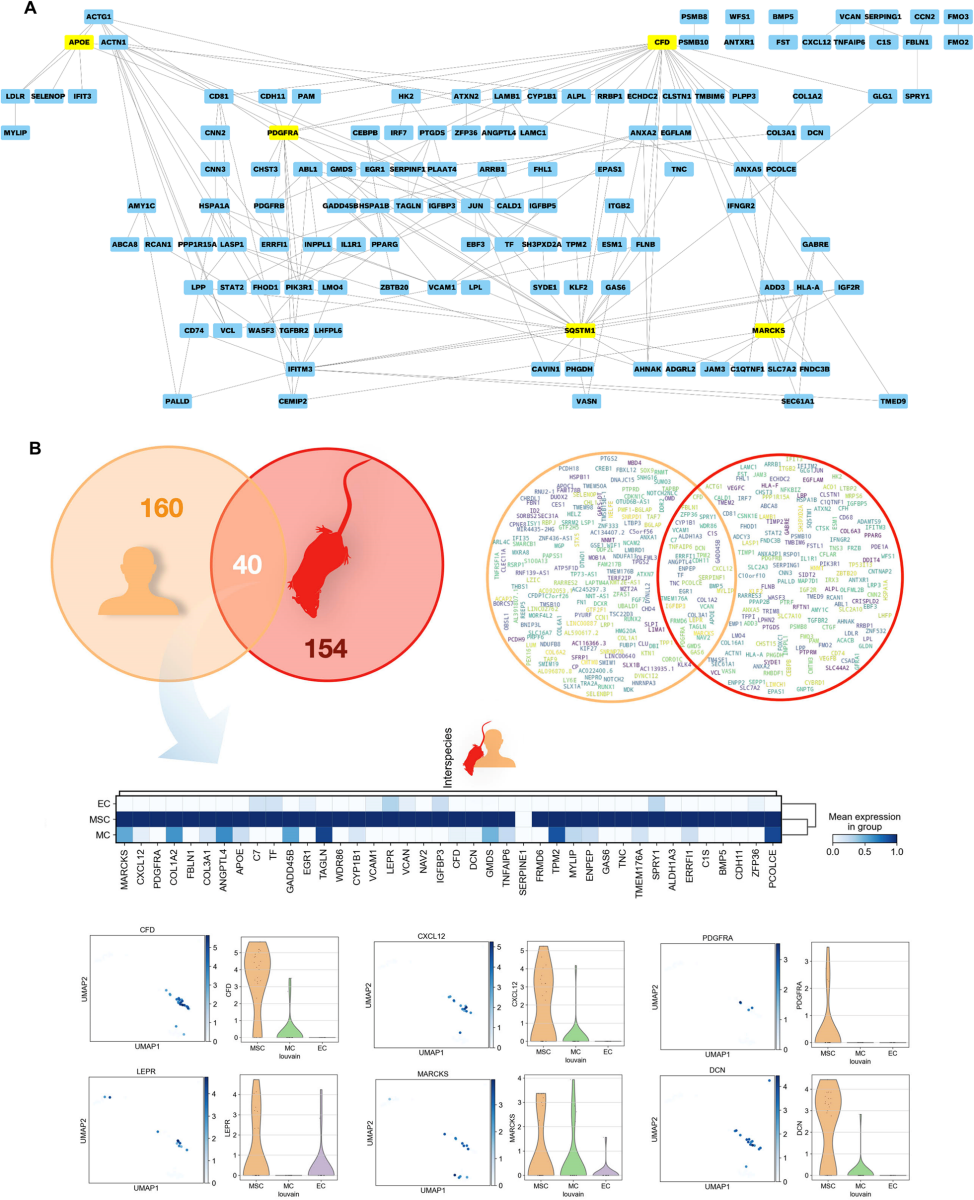
Key molecular factors specific to BM-MSCs. **A** Protein–protein interaction (PPI) network study revealed key factors that were the most connected to the others. Analysis performed with Cytoscape. **B** Venn diagram showing the number and the name of DEGs between human BM-MSCs analyzed above and mouse BM-MSCs previously described [26, 27, 29, 30]. Heatmap panel: distribution of pro-hematopoietic interspecies markers visualized in matrix plot among human non-hematopoietic subsets and mice identified above (size of dots are related to the percentage of positive cells and colors to gene normalized expression according to the color scale). Lower panel: expression of the interspecies (human and mouse) markers (*CFD*, *CXCL12*, *PDGFRA*) among non-hematopoietic human subsets in UMAP plots described above and quantified in violin plots labeling significantly MSCs (*p* < 0.05)

In addition, using topological features of protein–protein interaction (PPI) analysis, we determined more precisely key functions that are conserved in human native BM-MSCs. For this, we needed a training gene set and a test gene list. The training gene set was derived from the GSEA described above, and the test gene list contained our scRNA-seq data. As depicted in Fig. 3, we noted several hubs, in particular CFD, PDGFRA, SQSTM1, MARCKS and APOE (Fig. 3A). In addition, by analyzing the module network, the following biological processes were highlighted: positive regulation of fibroblast proliferation (PDGFRB, PDGFRA, ABL1, JUN, GAS6, CD74), skeletal system development (FST, BMP5, COL1A2, ALPL, VCAN, CDH11), angiogenesis (ACTG1, CYP1B1, CCN2, PDGFRB, EPAS1, CALD1, JUN, ESM1, ANXA2, ANGPTL4, JAM3), cell adhesion (CLSTN1, INPPL1, TNC, CYP1B1, CCN2, VCL, VCAM1, CXCL12, TNFAIP6, ITGB2, LAMC1, VCAN, CDH11, LAMB1), ageing (CCN2, PDGFRB, IGFBP5, SERPINF1, SERPING1, VCAM1, TGFBR2), organ regeneration (IGF2R, CXCL12, TGFBR2, GAS6), HSC migration (CXCL12), osteoclast regulation (CD81, SH3PXD2A), and adipogenic-fate regulation (ZFP36, LPL, PPARG, CEBPB) (see Additional file 9: Table S8).

Overall, human native BM-MSCs expressed a conserved core of genes related to biological functions. The independent definition of this core set from experimental procedures will help to further portray BM-MSCs with evident functions in skeletal biology, the HSC niche, vascular cell interactions and formation of ECM and tight adhesions to other cells or ECM.

### Conserved phenotype and functions of native BM-MSCs between humans and mice

Data describing the phenotype and functions of BM-MSCs in culture showed strong similarities within and between species, notably in humans versus mice. At the level of native cells, few papers reported common markers between these species. Notably, in previous studies, we isolated human and mouse native BM-MSCs by using a common marker [32, 60]. This study aimed to extract more common markers that should reflect inter-species shared functions supported by conserved molecular pathways. By using an integrated interspecies analysis (see Methods), we compared the expression of molecules and key markers of MSC populations between mice and humans (Fig. 3B). We obtained 37 common marker-genes (Fig. 3B) upregulated in MSCs (e.g., *VCAM1*, *LEPR*, *MGP*, *GAS6*, *CDH11*, *WIF1*, etc.). Three of them, *VCAM1*, *CXCL12* and *LEPR*, were previously reported as native MSC markers in humans and mice. Other genes were connected to ECM synthesis and structure: *FBLN1, COL3A1, VCAN, DCN, PCOLCE* and *TNC*. These molecules could contribute to structure the BM microenvironment sustaining hematopoiesis or bone formation. Genes regulating cell proliferation and differentiation (*MDK*, *IGFBP5*, *PTPRD*, *WIF1*, *EGR1*, *APOE*, *GAS6*) were also expressed in both species.

To explore MSC shared functions independent of the species of origin, we established a PPI network with the gene list described above. Several modules were found, notably EGR1, ZFP36, APOE, MARCKS, and PDGFRA (Additional file 10: Table S9). Besides the overall analyses of molecular networks, we examined TFs and DNA/RNA BPs governing BM-MSCs in humans and mice. By data deconvolution, we reduced the data to three modules: EGR1, ZFP36 and SPRY1 ((Additional file 1: Fig. S11). Module 1, EGR1, belongs to the EGR family of C2H2-type zinc-finger proteins and is shown to intervene in proliferation and differentiation processes, whereas module 2, ZFP36, concerned the cellular macromolecule catabolic process and regulation of mRNA processing. Module 3, SPRY1, contained genes connected to the regulation of extracellular signal-regulated kinase (ERK) mitogen-activated protein kinase (MAPK) pathways and circulatory system development (transmembrane receptor protein tyrosine kinase signaling pathway, epidermal growth factor receptor signaling pathway, cellular response to growth factor stimulus, regulation of MAPK cascade, vasculature development) (Additional file 10: Table S9). SPRY1 has also been shown to modulate adipocyte differentiation [61].

**Fig. 4:**
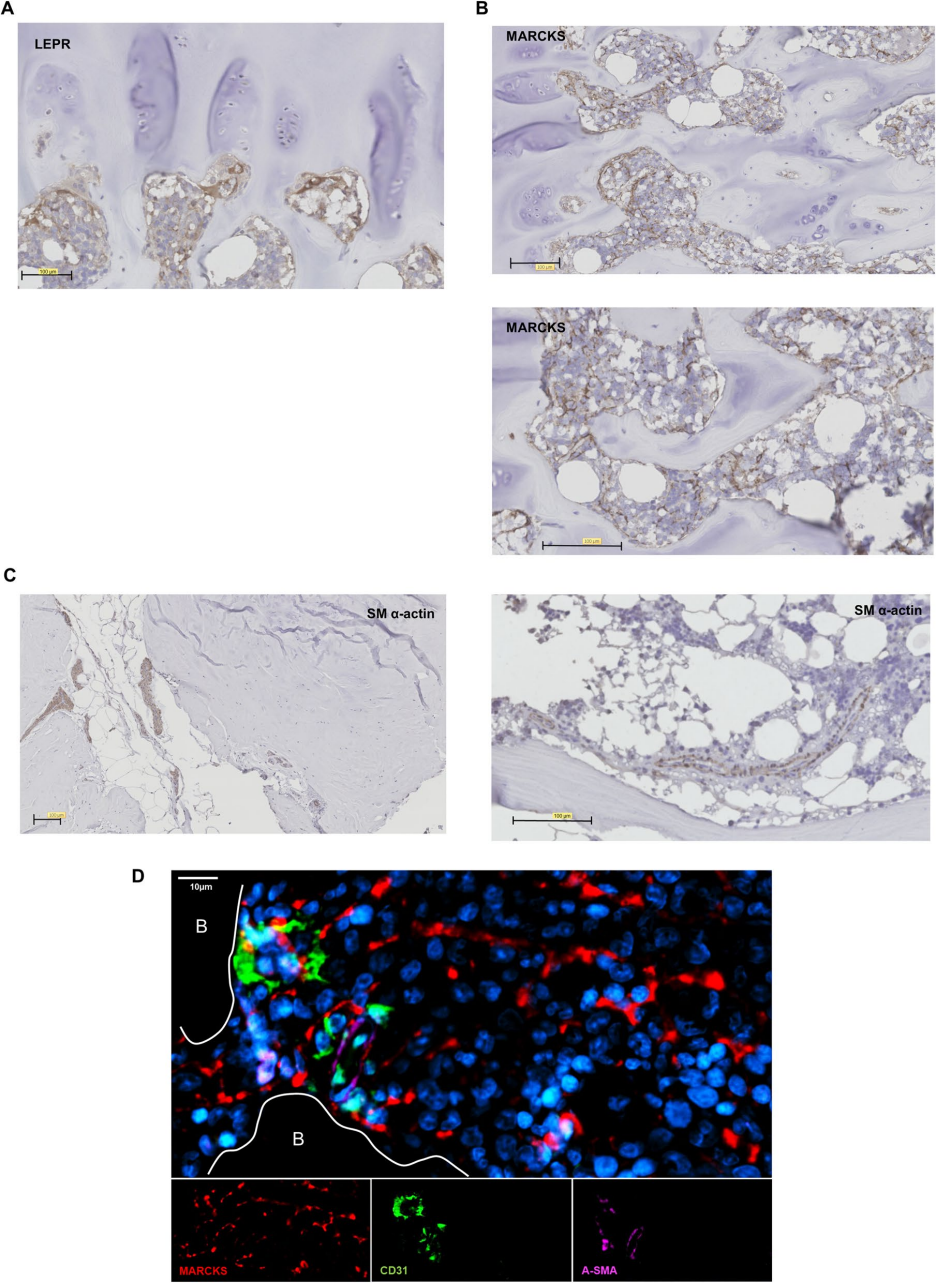
Histological staining of new BM-MSC markers. According to our data, micro slices of BM biopsies were labeled with anti-LEPR (**A**), anti-MARCKS (**B**), and smooth muscle α-actin (**C**) antibodies (*n* = 3). **D** Co-staining of MARCKS + MSCs, CD31 + ECs and ACTA2 + (α-SMA +) MCs by immunofluorescence. Nuclei were labeled by DAPI. White line defining trabecular bone (**B**). Scale bar 10 µm

Although these in silico analyses showed conserved core molecules, they needed confirmation by direct observation. Among markers, we were interested in MARCKS expressed in humans and mice, and because of its association with EGR1, a putative key TF found previously. We performed immunological staining of human BM biopsies; see Fig. 4. As a positive control, we labeled LEPR as an accepted native MSC marker. The expression of LEPR and MARCKS was observed in bone lining cells as well as in the vicinity of vessels or interspaced within the hematopoietic niche. Although more heterogeneous, the present histological characterization was compatible with the classical view of the MSC situation in the BM [62] (Fig. 4). In comparison, α smooth muscle actin (α-SMA or ACTA2) expression, defined as a marker of BM stromal cells [63], was restricted to vascular smooth muscle cells around the hematopoietic niches. Immunostaining of BM biopsies with anti-αSM actin antibodies marked only perivascular cells from arterioles and venules, which was in accordance with mRNA expression showing ACTA2 as characteristic of MCs but not MSCs. To complete the characterization, we used NHERF2 (SLC9A3R2), which specifically stains ECs (Additional file 1: Fig. S12), and marked a different type of cells as compared with LEPR or MARCKS staining. Finally, we combined antibodies for staining MSCs, ECs and MCs on the same BM biopsy slices. As shown in Fig. 4D, MARCKS + cells formed networks of stromal cells throughout BM. Of note that there were no overlaps between MARCKS, CD31 and α-SMA staining. This confirmed that MARCKS + MSCs were actually different from ECs and MCs as showed by scRNA-seq data. In summary, MARCKS protein may be used as MSC marker in association with EC and MC markers to phenotypically characterize the whole non-hematopoietic compartment of human BM (Additional file 11).

## Discussion

In the present study, we generated sets of data that served to fuel in silico analyses to profile BM-MSCs and to understand the non-hematopoietic cell heterogeneity in the human BM niche. In addition, it allowed for providing some characteristics of key molecular pathways and markers of the different cell types. More precisely, the data should help with a better understanding of the MSC compartment and its function.

Despite the restricted number of harvested cells and the inherent limitations of scRNA-seq, our multimodal studies enabled us to strengthen the relevance of our investigations. This approach further highlighted similarities in expression profiles of human BM-MSCs whatever the characterization methods and between humans and mice. The core genes shared by human and mouse cells certainly reflect key functions of BM-MSCs notably toward skeletal tissue or HSC niches.

We obtained a clear and reliable phenotype of MSCs with data from very recent reports using scRNA-seq for determining identities of cells constituting human tissues (*T. sapiens* [40] consortium and Ye et al. [45]) notably for application to MSCs, ECs and MCs. Of note, these reports used cells from both sexes (male and female), which reinforces the relevance of phenotypes and functions for BM cells. In addition to these scRNA-seq inputs, we used others obtained from different high-throughput and cell selection strategies. Furthermore, to our multimodal approach, we added characterization data of mouse BM-MSCs. Taken together, this analysis resulted in a description of markers and functions that should be common to adult BM-MSCs whatever the sex or species (humans or mice).

As expected, these genes contribute to functions revealing BM-MSC physiological roles (i.e., the formation of all bone cells, regulation of the vascular tree, organization of ECM proteins, cell adhesion and migration). In addition, BM-MSCs are transcriptionally primed for the HSC niche construction (e.g., via CXCL12 expression). Among pathways that emerged was an adipocyte trait for BM-MSCs. Given the age of the BM donors we included in the study, it was not surprising to find an adipogenic signature, because BM becomes more adipose with age, forming marrow adipose tissue [64]. This latter feature, also described in aged mouse models, was shown to be reversible when stress in hematopoiesis arises or upon anti-adipogenic stimulation. Among the adipogenic genes induced in aged MSCs, CFD and APOE levels were enhanced, in accordance with our results. Although APOE is commonly described for adipose cells, the adipokine CFD, also called adipsin, is less known. Adipsin is a protease that catalyzes the cleavage of factor B, the rate-limiting step of the alternative pathway of complement activation. This protein also regulates insulin secretion in mice [65] and was recently found upregulated in adipose BM during aging [66]. Knock out of adipsin gene in mice specifically suppressed the adiposity of BM but not peripheral adipose depots, with an improvement of bone mass during aging. Therefore, CFD could be a marker of MSCs from marrow adipose tissue, and its regulation could be modulated to improve bone defects due to aging or metabolic disease.

Besides identifying well-known markers of MSCs, we revealed three modules consisting of inter-species common factors that might be at the center of key pathways: EGR1, ZFP36 and SPRY1. EGR1 regulates numerous genes intervening in mitogenesis and differentiation processes and mediates responses to hypoxia. It is also involved in inflammatory processes during development or after tissue injuries. These overall mechanisms are basically described for MSCs. Interestingly and additionally, EGR1 was very recently shown to be involved in HSC niche and its expression by BM stromal cells was associated with hematopoietic supporting genes [49]. Therefore, EGR1 should be activated when human and mouse MSCs are engaged in differentiation processes, in the modulation of inflammation or for regulation of hematopoiesis for instance. ZFP36 is an mRNA decay activator involved in cellular responses to cytokine or growth factor stimulus notably to modulate pro-inflammatory signals. ZFP36 is expressed early in the adipogenic lineage and can regulate pro-inflammatory cytokine expression [67]. This finding agrees with the adipose feature of MSCs we found. Finally, SPRY1 can inhibit the ERK1/2-MAPK pathway. SPRY1 level was found increased during early-onset adipocyte differentiation when the master TF adipogenesis CCAAT/enhancer-binding protein β (C/EBP β) is needed [68]. Therefore, in human adult BM, MSCs exhibit an adipogenic commitment probably in response to aging. These key factors could be used to modulate this fate for improving bone strength and hematopoiesis in older people.

The combination of different datasets and relationship studies with PPI suggested some other molecules as hubs for a large panel of MSC functions. Example molecules are APOE, CFD (described above), PDGFRA, SQSTM1, and MARCKS. PDGFRA is a native MSC marker in mice and even in humans. It is used to typify MSCs in different tissues besides BM such as adipose tissue, skin, and muscle. Therefore, PDGFRA is a pan-native MSC marker. Culture processing, largely used to expand MSCs, induces a profound decrease in its expression. We then focused on MARCKS for several reasons described above. MARCKS is also found as a hub in gene sets from an in silico study of numerous cultured and native mouse BM stromas [60]. Therefore, MARCKS should have crucial functions in BM-MSCs, which needs to be clarified. MARCKS is highly conserved in vertebrates because of its central role in nervous system. MARCKS binds to internal cytoplasmic membrane and promotes cross-linking of actin microfilaments, and upon phosphorylation, it translocates to the cytosol. These different situations in the cell allow MARCKS to modulate the migration of cells and vesicular trafficking. This protein intervenes in developmental processes and tissue regeneration. We found MARCKS highly expressed in native BM-MSCs, and immuno-histology staining showed strong expression in phenotypically reticular cells that could be associated with MSCs in accordance with the similar labeling by LEPR antibodies. This feature was also seen when BM was stained for CD271 expression, a well-known native MSC marker [69]. MARCKS + cells are located from the abluminal position of vessels to trabecular bone. They also exhibit long cytoplasmic protrusions, a morphology similar to that described for abluminal reticular cells, MSC-like cells. Therefore, we propose MARCKS as a new native MSC marker with a function that has to be yet defined.

In addition to MSCs, we described ECs and MCs. ECs are equally important in supporting HSC migration, long-term survival, differentiation and homing. Notably, the endothelium intervenes in niche functions by acting directly on the stromal cells involved in the control of HSCs [70]. We propose new markers of BM ECs with key molecules modulating their functions. For instance, we detected KLF4 and KLF2 in BM ECs. These TFs are well-known flow-sensitive TFs. The blood flow in vessels may activate these flow-sensitive TFs, which in turn can induce inflammation in ECs. We also found STAT3 expression in these types of cells. STAT3 regulates numerous molecular pathways, notably IL6 during inflammation. These different traits prompted us to propose that the BM ECs described in the present study could be associated with ECs in the inflammation context such as during aging (the inflammaging context) in agreement with the adiposity of aged BM found here.

The last non-hematopoietic cells that we isolated were MCs. We identified a panel of markers that could be characteristic of BM-MSCs. These cells were historically associated with MSCs until a recent paper showed that Tbx18 + pericytes are not MSCs [71]. Nevertheless, MC is general term encompassing pericytes and abluminal perivascular cells such as adventitial cells; the latter might be at the origin of MSCs [72]. A deeper scRNA-seq performed specifically on these populations should be informative to clarify their relationship. The PPI studies for MCs gave little information but underlined the expression of YBX1, a DNA and RNA binding protein, and NR2F2. These factors were described to intervene in molecular pathways in pericytes for regulating specific MC functions for their contractility or in angiogenesis processes [73, 74]. MCs also featured a panel of cytoskeleton proteins and growth factors dedicated to interact with ECs for the integrity of functional vessels.

## Conclusion

In conclusion, our single-cell dataset and our multimodal studies may serve as a molecular and cellular blueprint of the transcriptional states of BM-MSCs and their relation with ECs and MCs constituting the other non-hematopoietic BM compartment. The new markers we propose may also be used for deeper characterization studies, their direct visualization and their exploration at functional levels. Our results also highlight the phenotypic and functional states of MSCs in the adult aged environment, which may facilitate studies seeking to address bone and hematopoietic defects due to aging. Finally, because MSCs are used worldwide in numerous therapeutic protocols, a better characterization of these cells should help in collecting them from biological samples and improving the quality and efficiency controls for clinical use in regenerative medicine. Improving single-cell techniques by spatial transcriptomics with combined large-scale proteomic and lipidomic approaches offers promising perspectives to answer fundamental questions on the physiological dynamics of the BM. Furthermore, analyzing the changes in non-hematopoietic BM cells during hematopoietic malignancies, immunodeficiency, and aging would provide valuable information for developing curative strategies.

### Supplementary Information

**Additional file 1**. Supplementary Figures 1–12

**Additional file 2. Table S1:** Differentially expressed genes of all populations found in scRNA-seq experiments.

**Additional file 3. Table S2:** GO-terms of biological processes for EC population. n/a: not applicable.

**Additional file 4. Table S3:** GO-terms of biological processes for MC population. n/a: not applicable.

**Additional file 5. Table S4:** GO-terms of biological processes for MSC population. n/a: not applicable.

**Additional file 6. Table S5:** Signaling pathways activated in MSCs. n/a: not applicable.

**Additional file 7. Table S6:** Gene Set Enrichment Analysis between gene sets from scRNA-seq data and CD271+/CD200+ selected MSCs.

**Additional file 8. Table S7:** GO-terms of universal human MSCs.

**Additional file 9. Table S8:** GO-terms of biological processes from calculated module networks for MSCs.

**Additional file 10. Table S9:** Gene module networks common for human and mouse MSCs.

**Additional file 11. ** Supplementary figure legends.

## Data Availability

All transcriptomic data are available in GEO under the following references: GSE198049.

## Abbreviations

BM MSCs: Bone marrow mesenchymal stromal cells
ECs: Endothelial cells
MCs: Mural cells
scRNA-seq: Single-cell RNA-sequencing
HSCs: Hematopoietic stem cells
CFU-Fs: Colony forming unit-fibroblasts
TFs: Transcription factors
ECM: Extracellular matrix
PBS: Phosphate buffered saline
DMSO: Dimethylsulfoxide
UMAP: Uniform manifold approximation and projection
DEG: Differentially expressed genes
GO-term: Gene ontology-term
PPI: Protein–protein interaction
