# Supplementary material for: Single-cell RNA sequencing of human non-hematopoietic bone marrow cells reveals a unique set of inter-species conserved biomarkers for native mesenchymal stromal cells
Source: Stem Cell Res Ther. 2023 Aug 30;14:229. doi: 10.1186/s13287-023-03437-x (PMC10469496; doi:10.1186/s13287-023-03437-x)

Figure S1

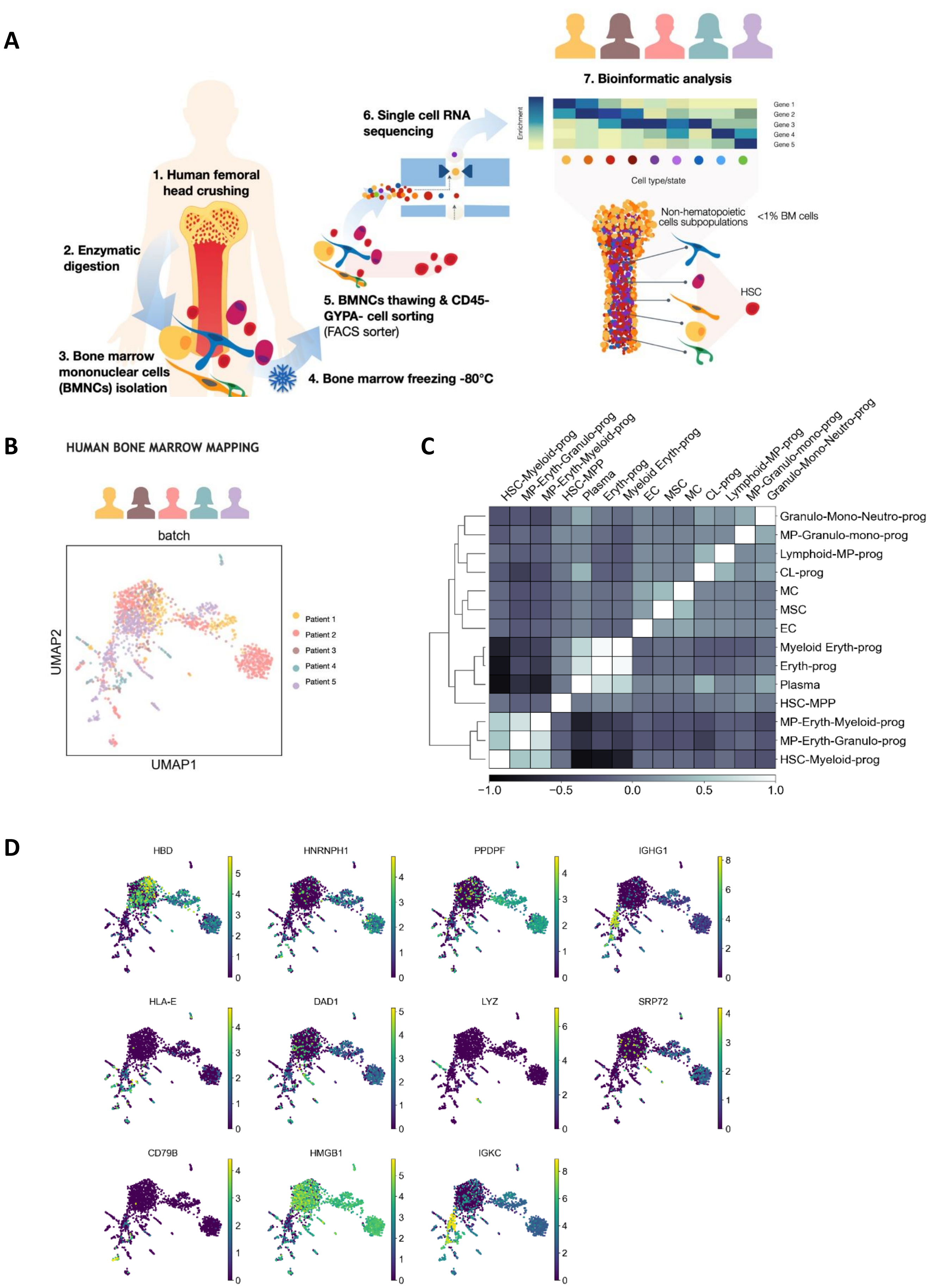

A
1. Human femoral head crushing
2. Enzymatic digestion
3. Bone marrow mononuclear cells (BMNCs) isolation
4. Bone marrow freezing -80°C
5. BMNCs thawing & CD45- GYPA- cell sorting (FACS sorter)
6. Single cell RNA sequencing
7. Bioinformatic analysis
Enrichment
Gene 1
Gene 2
Gene 3
Gene 4
Gene 5
Cell type/state
Non-hematopoietic cells subpopulations
<1% BM cells
HSC
B
HUMAN BONE MARROW MAPPING
batch
UMAP2
UMAP1
Patient 1
Patient 2
Patient 3
Patient 4
Patient 5
C
HSC-Myeloid-prog
MP-Eryth-Granulo-prog
MP-Eryth-Myeloid-prog
HSC-MPP
Plasma
Eryth-prog
Myeloid Eryth-prog
EC
MSC
MC
CL-prog
Lymphoid-MP-prog
MP-Granulo-mono-prog
Granulo-Mono-Neutro-prog
−1.0
−0.5
0.0
0.5
1.0
D
HBD
HNRNPH1
PPDPF
IGHG1
HLA-E
DAD1
LYZ
SRP72
CD79B
HMGB1
IGKC

Figure S2

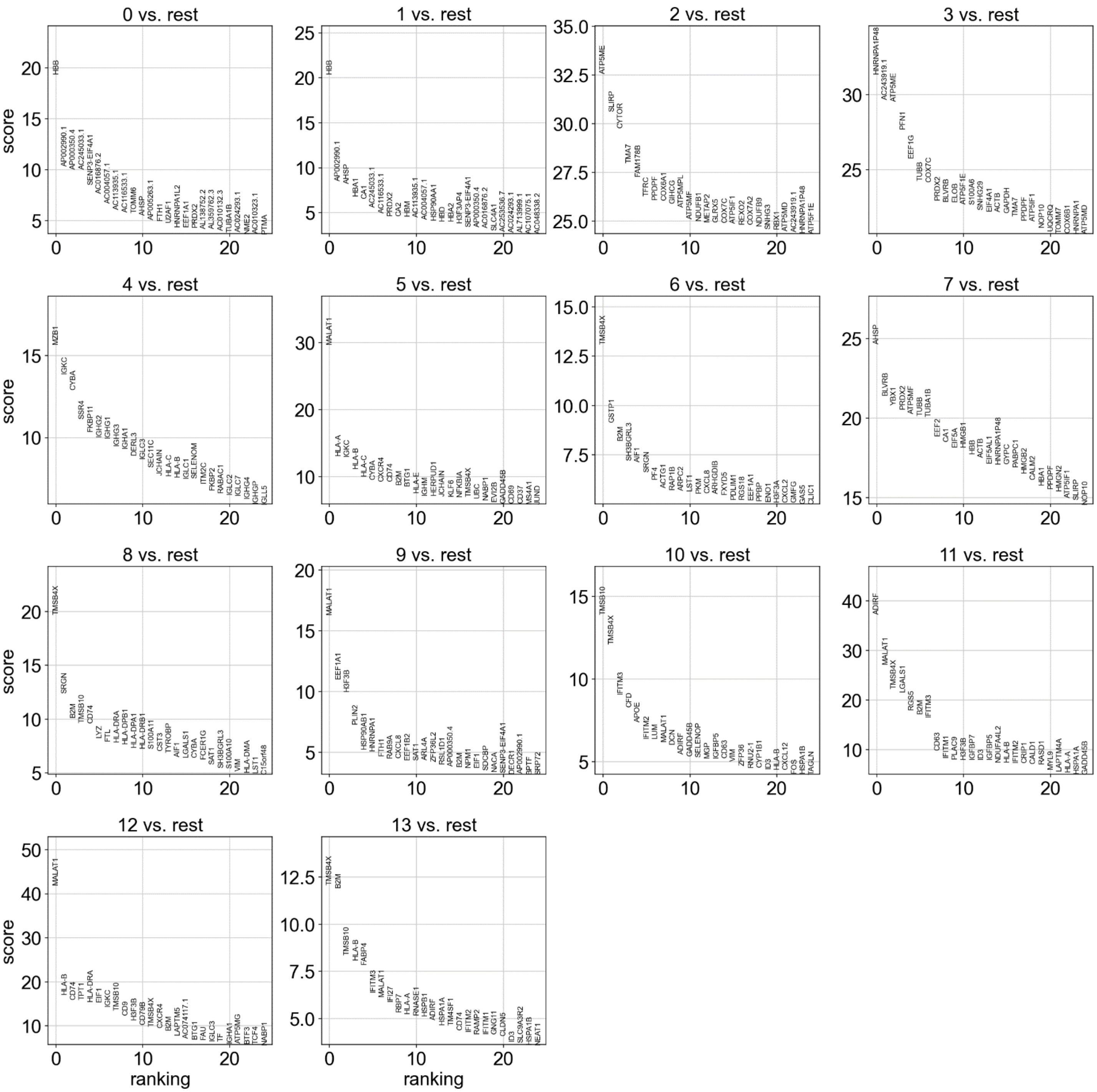
0 vs. rest
HBB AP002990.1 AP000350.4 AC245033.1 SENP3-EIF4A1 AC016876.2 AC004057.1 AC113935.1 AC116533.1 TOMM6 AHSP AP005263.1 FTH1 U2AF1 HNRNPA1L2 EEF1A1 PRDX2 AL138752.2 AL359762.3 AC010132.3 TUBA1B AC024293.1 NME2 AC010323.1 PTMA
1 vs. rest
HBB AP002990.1 AHSP HBA1 CA1 AC245033.1 AC116533.1 PRDX2 CA2 HBM AC113935.1 AC004057.1 HSP90AA1 HBD HBA2 H3F3AP4 SENP3-EIF4A1 AP000350.4 AC016876.2 SLC4A1 AC253536.7 AC024293.1 AL713999.1 AC107075.1 AC048338.2
2 vs. rest
ATP5ME SLIRP CYTOR TMA7 FAM178B TFRC PPDPF COX6A1 GIHCG ATP5MPL ATP5MF NDUFB1 METAP2 GLRX5 COX7C ATP5IF1 REXO2 COX7A2 NDUFB9 SNHG3 RBX1 ATP5MD AC243919.1 HNRNPA1P48 ATP5F1E
3 vs. rest
HNRNPA1P48 AC243919.1 ATP5ME PFN1 EEF1G TUBB COX7C PRDX2 BLVRB ELOB ATP5F1E S100A6 SNHG29 EIF4A1 ACTB GAPDH TMA7 PPDPF ATP5IF1 NOP10 UQCRQ TOMM7 COX6B1 HNRNPA1 ATP5MD
4 vs. rest
MZB1 IGKC CYBA SSR4 FKBP11 IGHG2 IGHG1 IGHG3 IGHA1 DERL3 IGLC3 SEC11C JCHAIN HLA-C HLA-B IGLC1 SELENOM ITM2C FKBP2 RABAC1 IGLC2 IGLC7 IGHG4 IGHGP IGLL5
5 vs. rest
MALAT1 HLA-A IGKC HLA-B HLA-C CYBA CXCR4 CD74 B2M BTG1 HLA-E IGHM HERPUD1 JCHAIN KLF6 NFKBIA TMSB4X UBC NABP1 EVI2B GADD45B CD69 CD37 MS4A1 JUND
6 vs. rest
TMSB4X GSTP1 B2M SH3BGRL3 AIF1 SRGN PF4 ACTG1 RAP1B ARPC2 LST1 PKM CXCL8 ARHGDIB FXYD5 PDLIM1 RGS18 EEF1A1 PPBP ENO1 H3F3A CXCL2 GMFG GAS5 CLIC1
7 vs. rest
AHSP BLVRB YBX1 PRDX2 ATP5MF TUBB TUBA1B EEF2 CA1 EIF5A HMGB1 HBB ACTB EIF5AL1 HNRNPA1P48 GYPC PABPC1 HMGB2 CALM2 HBA1 PPDPF HMGN2 ATP5IF1 SLIRP NOP10
8 vs. rest
TMSB4X SRGN B2M TMSB10 CD74 LYZ FTL HLA-DRA HLA-DPB1 HLA-DPA1 HLA-DRB1 S100A11 CST3 TYROBP AIF1 LGALS1 CYBA FCER1G SAT1 SH3BGRL3 S100A10 VIM HLA-DMA LST1 C15orf48
9 vs. rest
MALAT1 EEF1A1 H3F3B PLIN2 HSP90AB1 HNRNPA1 FTH1 RAB9A CXCL8 EEF1B2 SAT1 ARL4A ZFP36L2 RSL1D1 AP000350.4 B2M NPM1 EIF1 SDCBP NACA SENP3-EIF4A1 DECR1 AP002990.1 BPTF SRP72
10 vs. rest
TMSB10 TMSB4X IFITM3 CFD APOE IFITM2 LUM MALAT1 DCN ADIRF GADD45B SELENOP MGP IGFBP5 CD63 VIM ZFP36 RNU2-1 CYP1B1 ID3 HLA-B CXCL12 FOS HSPA1B TAGLN
11 vs. rest
ADIRF MALAT1 TMSB4X LGALS1 RGS5 B2M IFITM3 CD63 IFITM1 PLAC9 H3F3B IGFBP7 ID3 IGFBP5 NDUFA4L2 HLA-B IFITM2 CRIP1 CALD1 RASD1 MYL9 LAPTM4A HLA-A HSPA1A GADD45B
12 vs. rest
MALAT1 HLA-B CD74 TPT1 HLA-DRA EIF1 IGKC TMSB10 CD9 H3F3B CD79B TMSB4X CXCR4 B2M LAPTM5 AC074117.1 BTG1 FAU IGLC3 TF IGHA1 ATP5MG BTF3 TCF4 NABP1
13 vs. rest
TMSB4X B2M TMSB10 HLA-B FABP4 IFITM3 MALAT1 IFI27 RBP7 HLA-A RNASE1 HSPB1 ADIRF HSPA1A TM4SF1 CD74 IFITM2 RAMP2 IFITM1 GNG11 CLDN5 ID3 SLC9A3R2 HSPA1B NEAT1
score
ranking

Figure S3

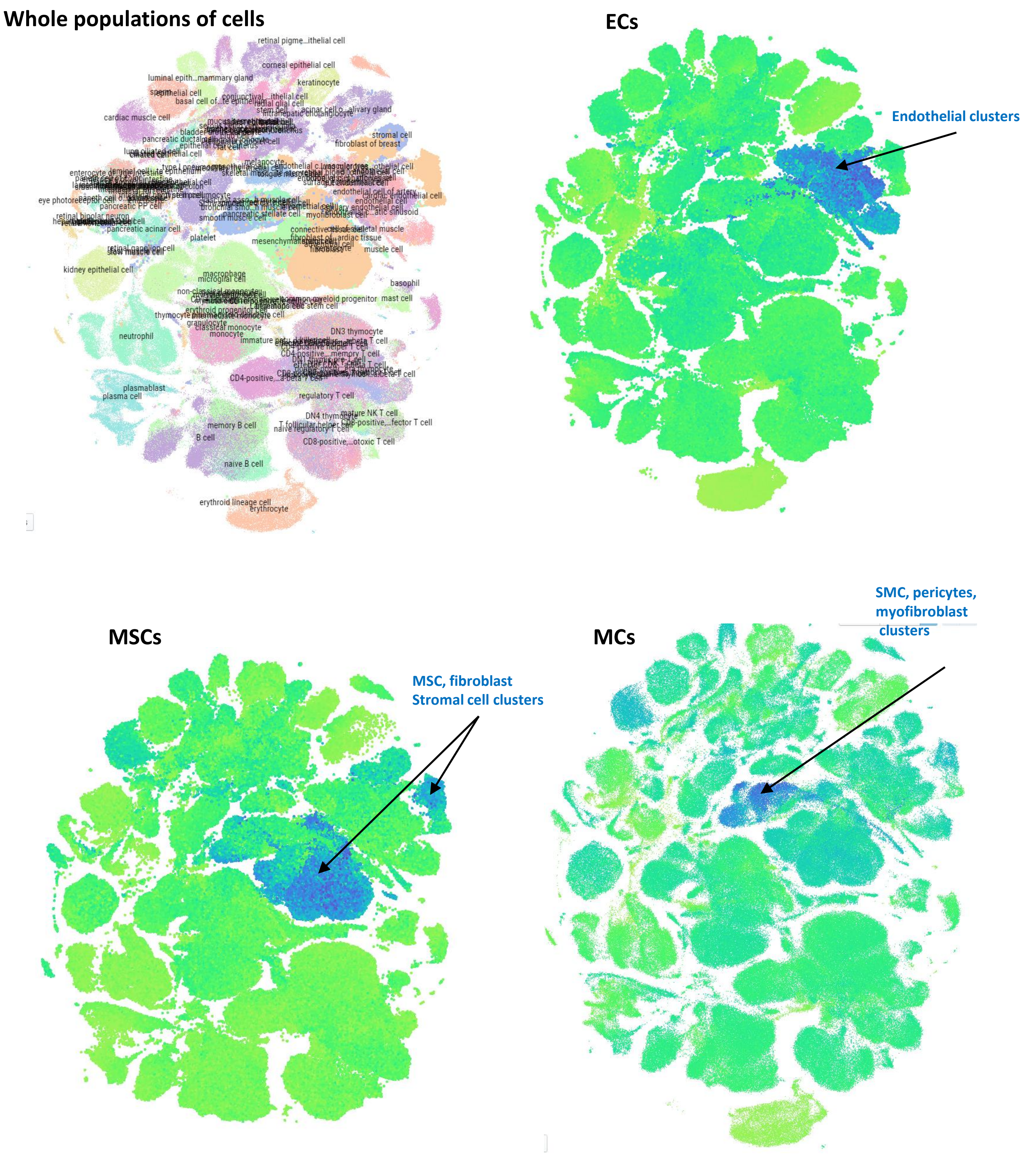

(Tabula Sapiens, Jones et al. 2022)

Figure S4

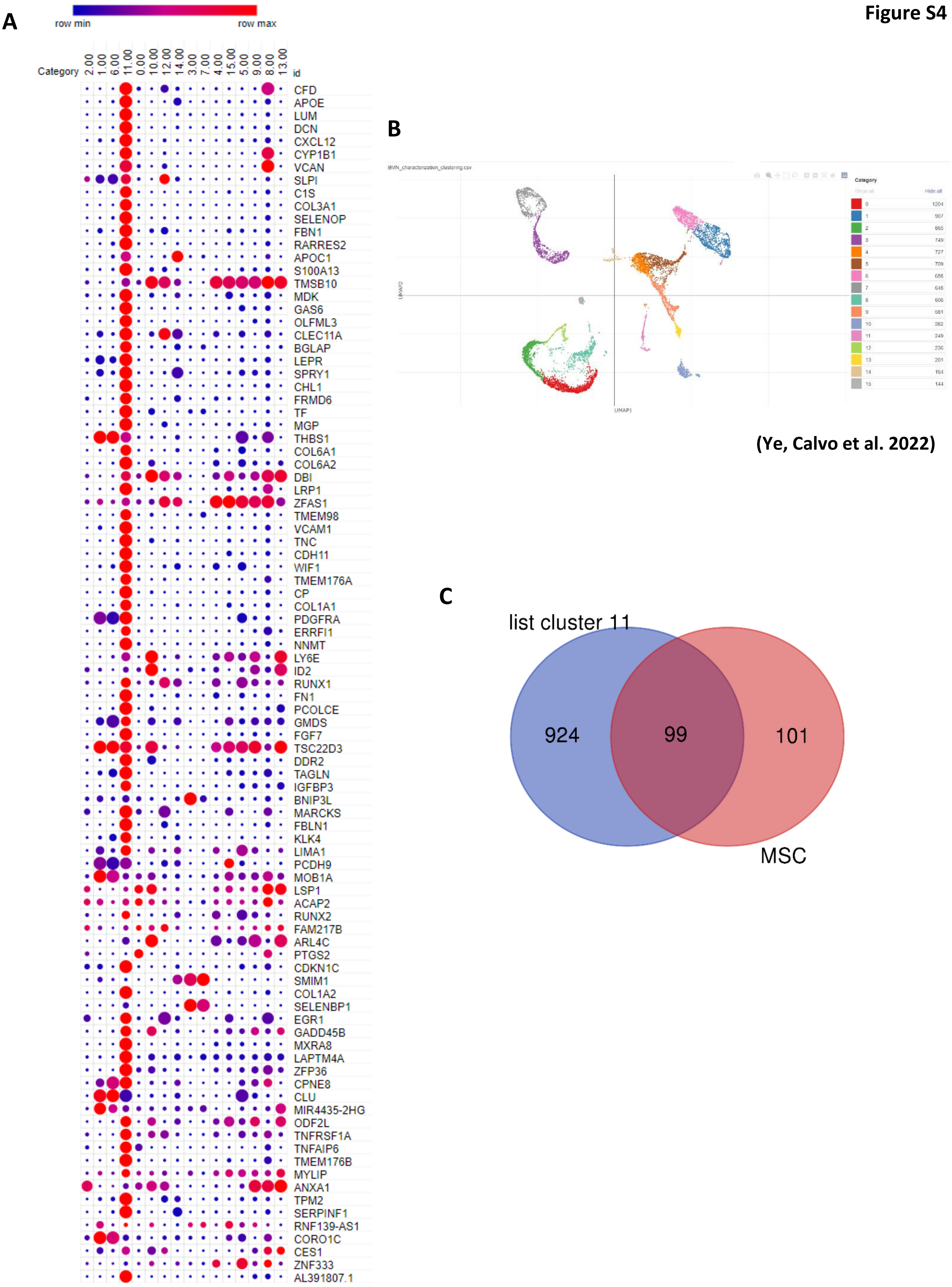

(Ye, Calvo et al. 2022)

Figure S5

Osteogenesis

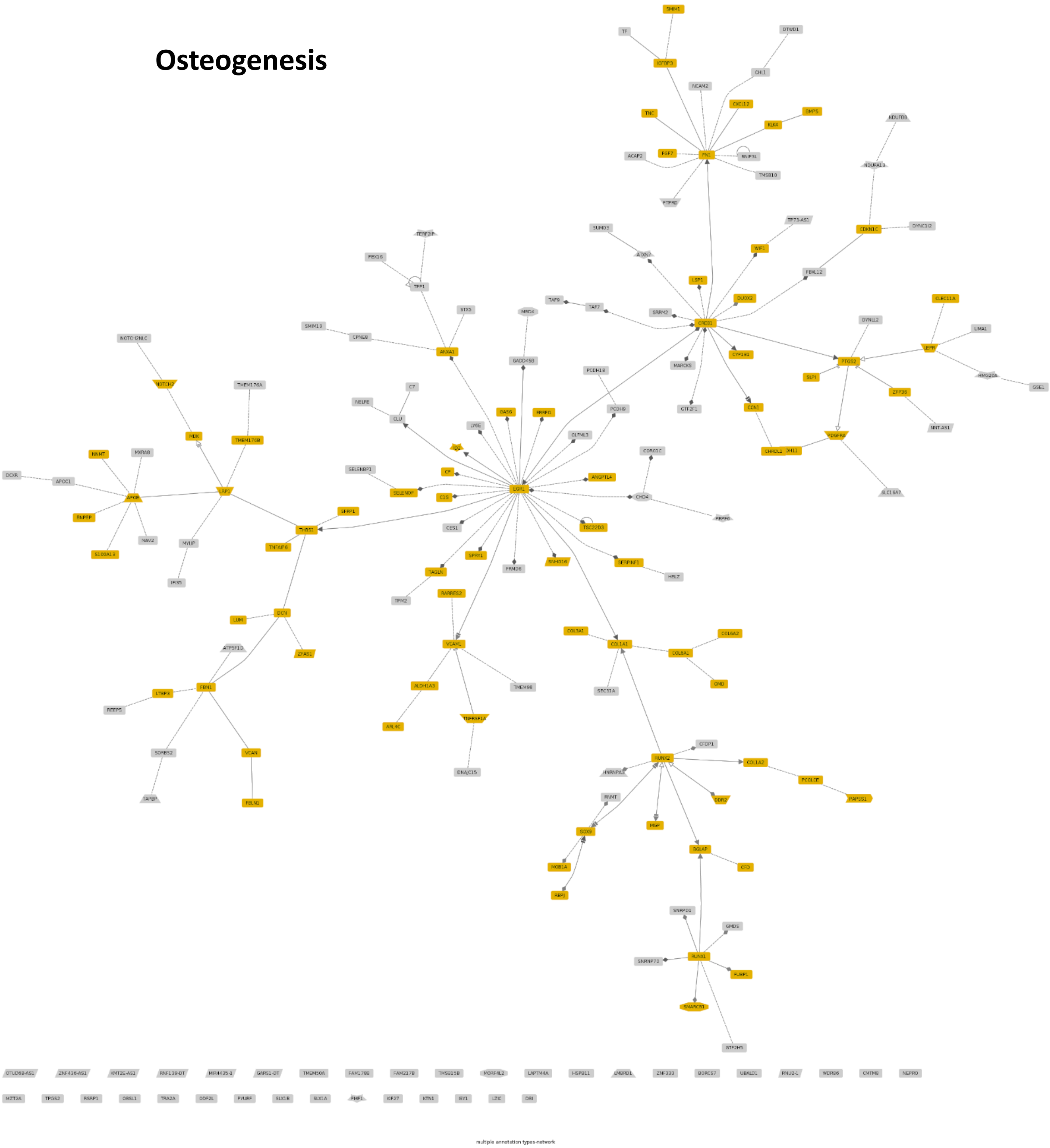

Figure S6

Adipogenesis

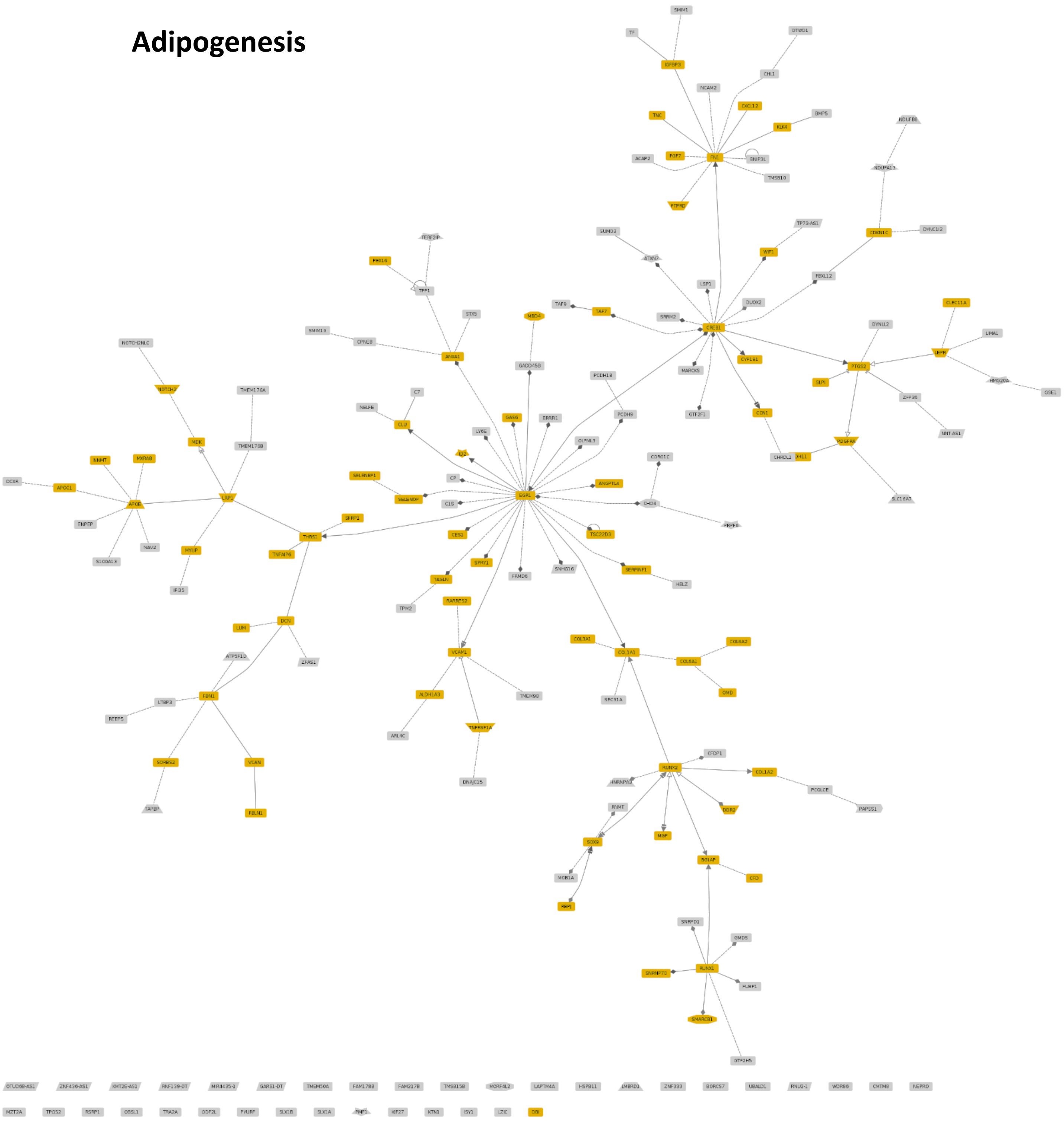

multiple annotation types network

Figure S7

Hematopoiesis

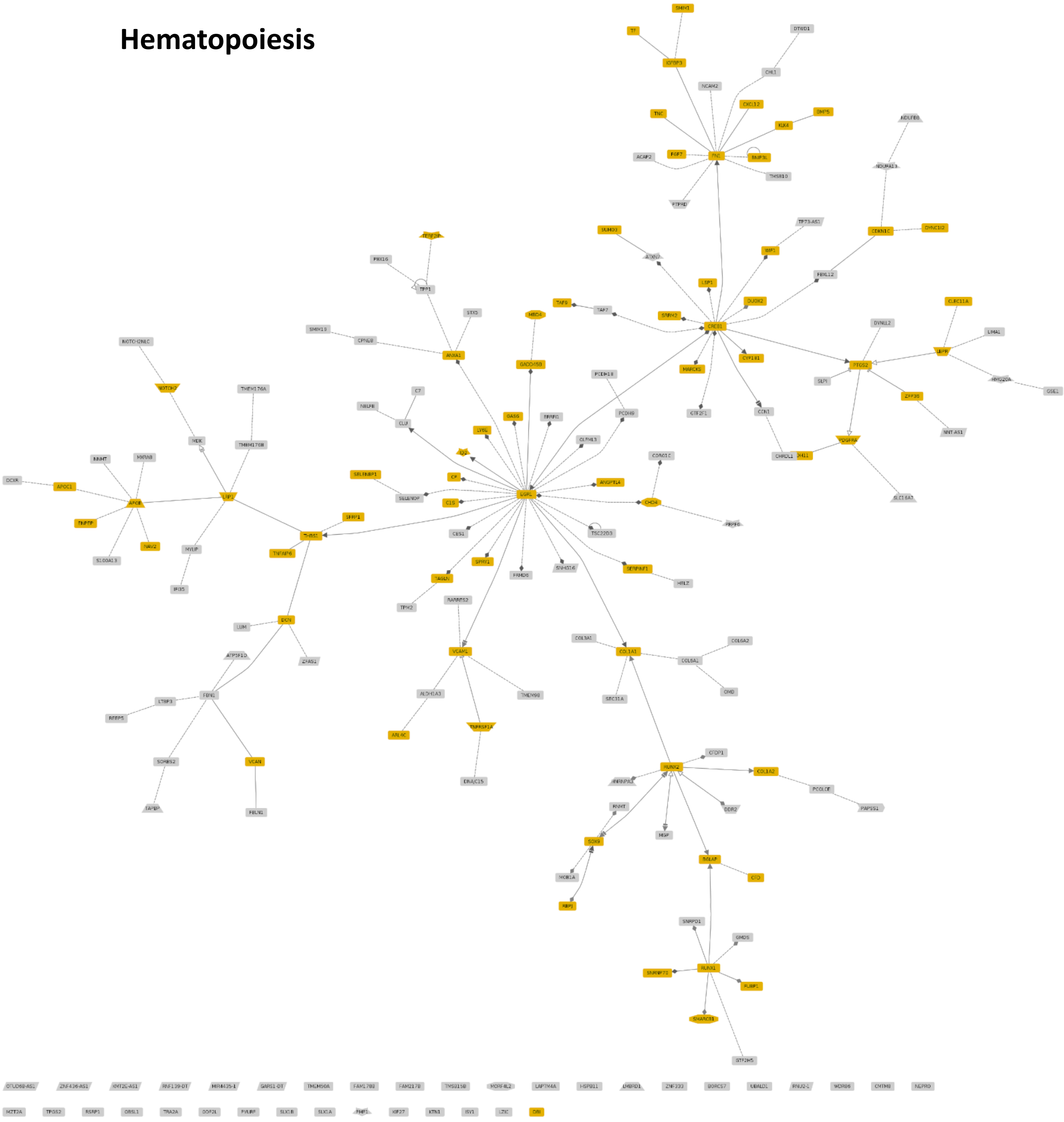

multiple annotation types network

Figure S8

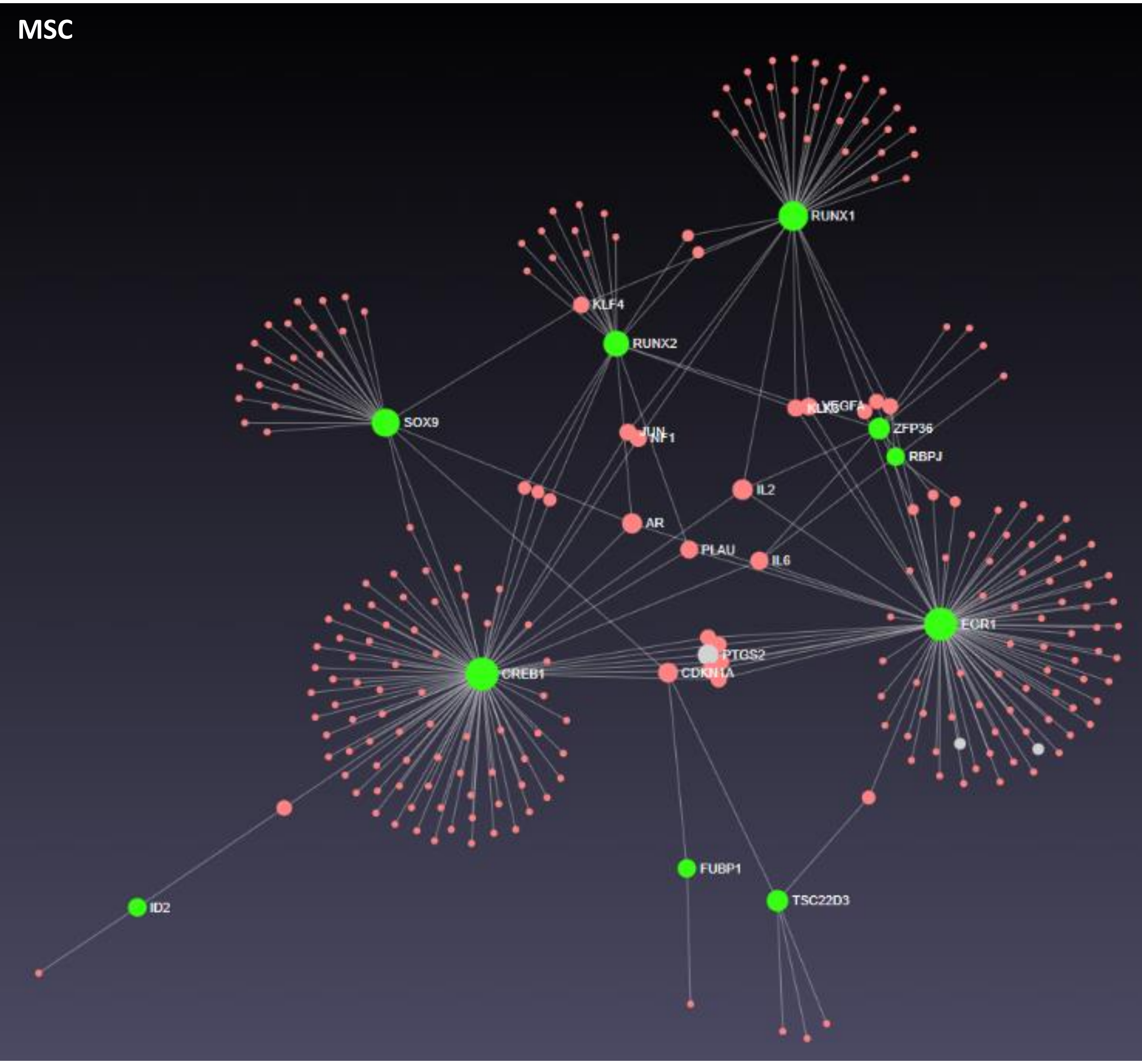
MSC
RUNX1
KLF4
RUNX2
SOX9
VEGFA
JUN
NF1
ZFP36
RBPJ
IL2
AR
PLAU
IL6
EGR1
PTGS2
CDKN1A
CREB1
FUBP1
TSC22D3
ID2

Figure S9

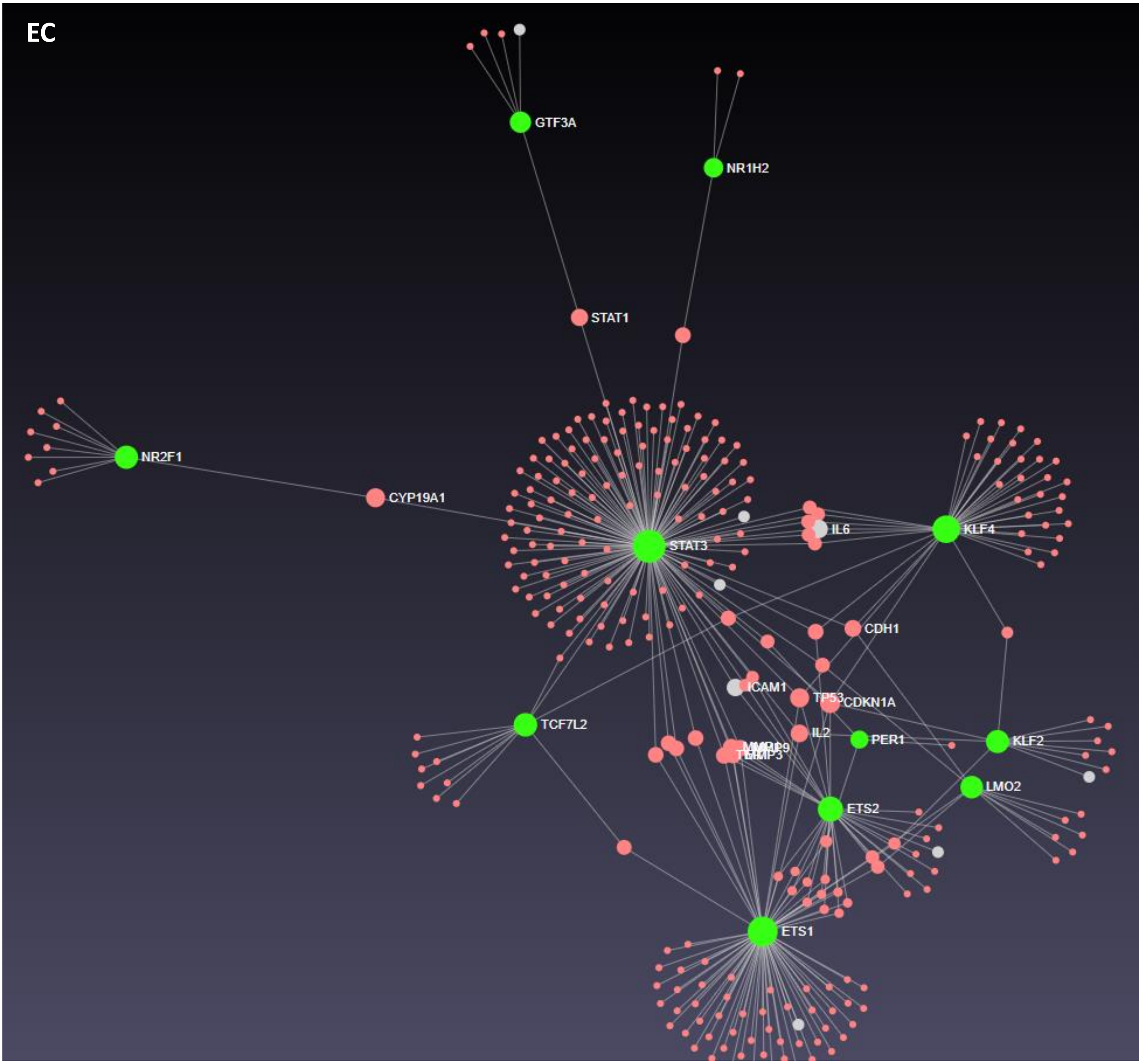
EC
GTF3A
NR1H2
STAT1
NR2F1
CYP19A1
STAT3
IL6
KLF4
CDH1
ICAM1
TP53
CDKN1A
TCF7L2
IL2
PER1
KLF2
LMO2
ETS2
ETS1

Figure S10

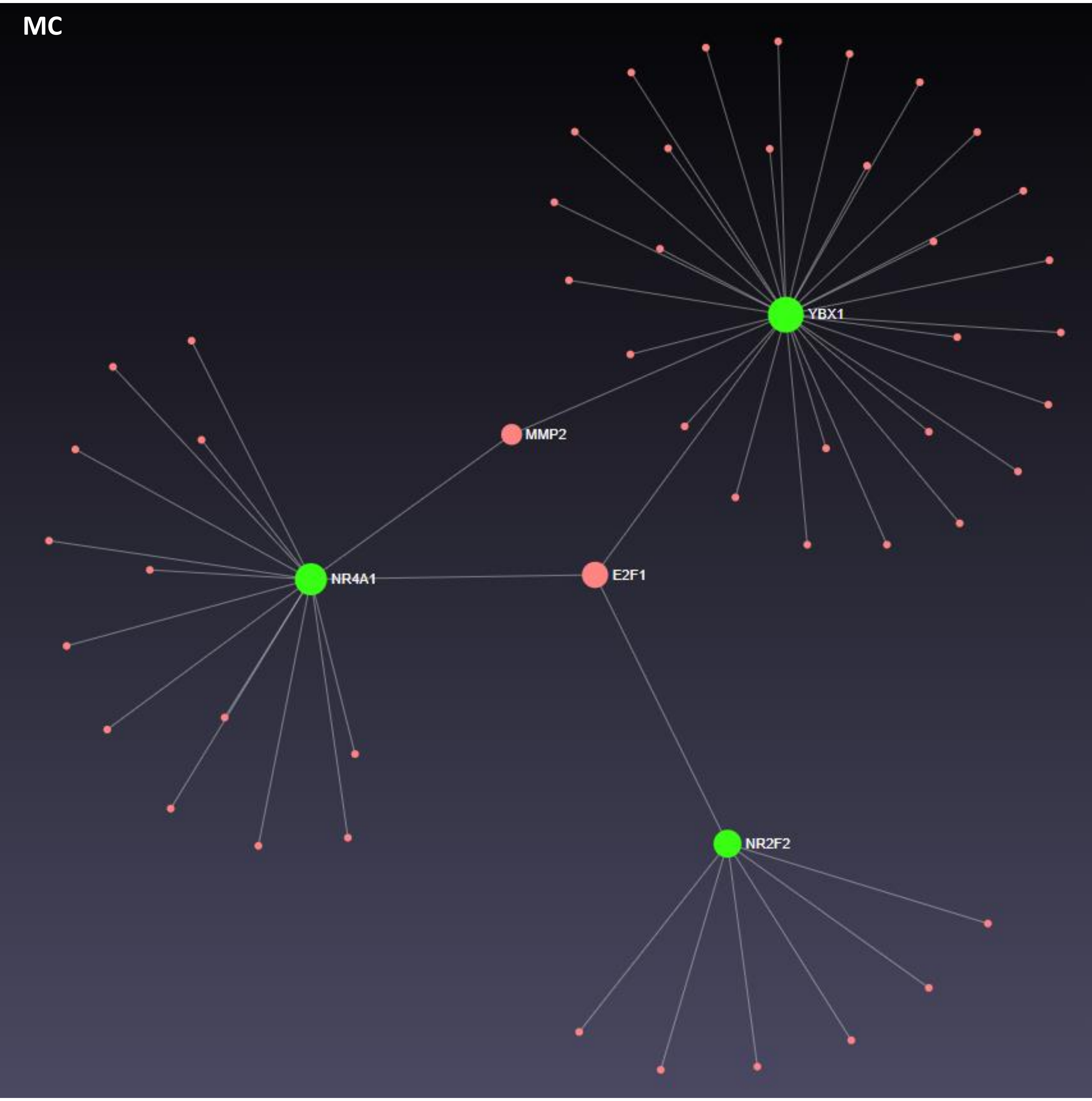

Figure S11

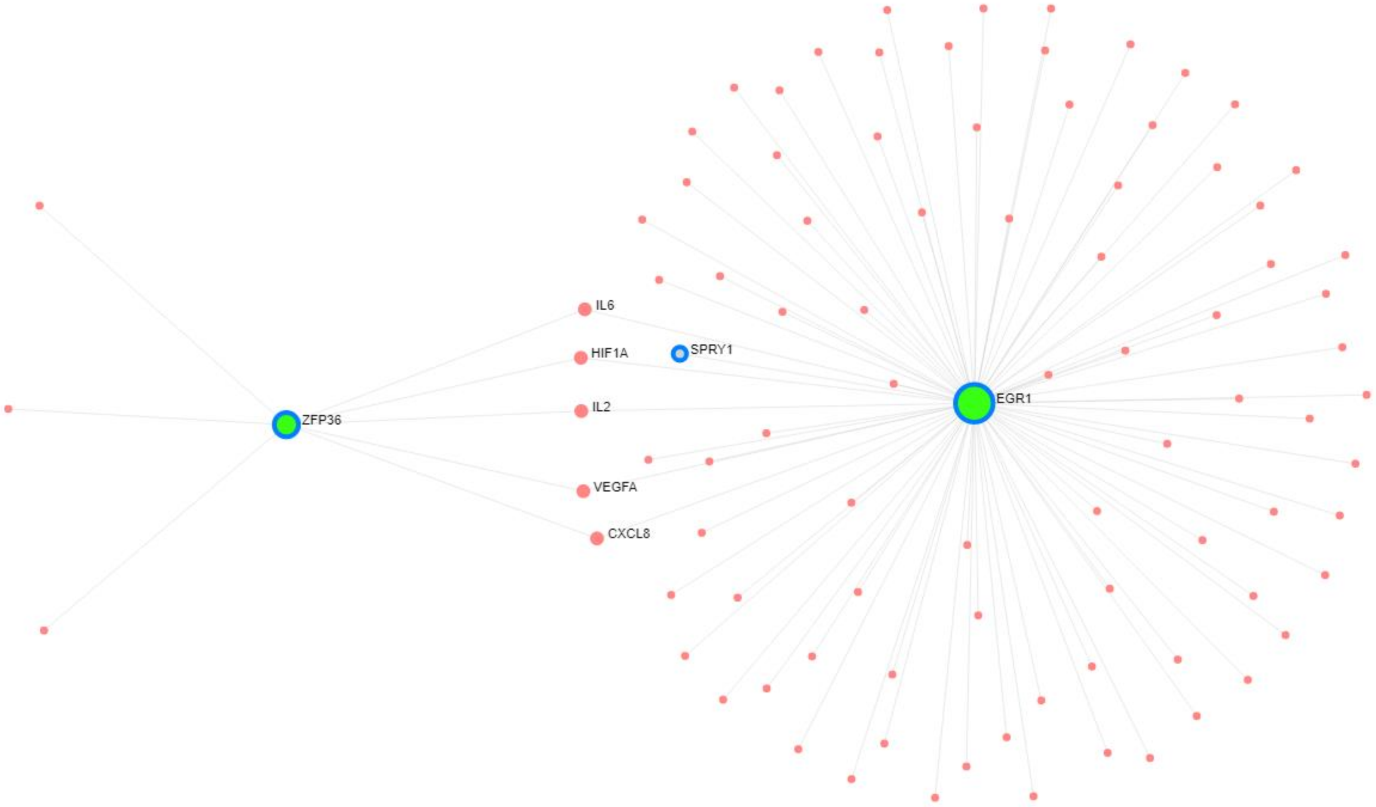

Figure S12

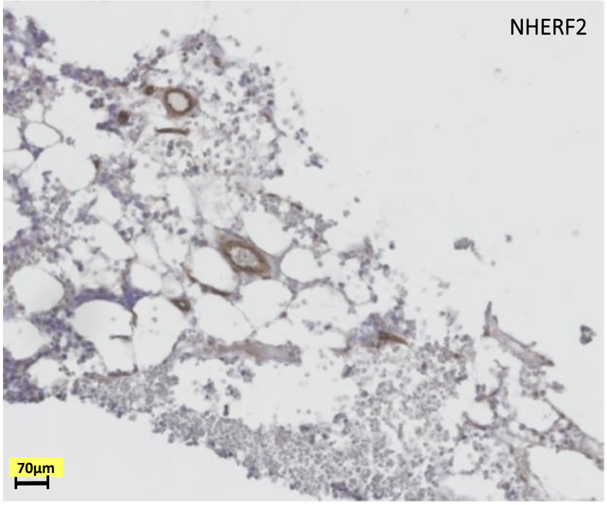

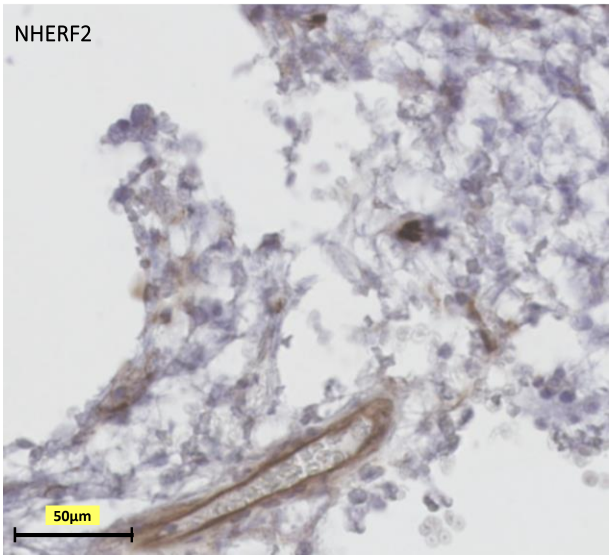

Supplement: Supplementary file 1 — Additional file 1. Supplementary Figures 1–12 [file 13287_2023_3437_MOESM1_ESM.pdf]
