# Supplementary material for: Single-cell RNA sequencing of human non-hematopoietic bone marrow cells reveals a unique set of inter-species conserved biomarkers for native mesenchymal stromal cells
Source: Stem Cell Res Ther. 2023 Aug 30;14:229. doi: 10.1186/s13287-023-03437-x (PMC10469496; doi:10.1186/s13287-023-03437-x)
Supplement: Supplementary file 11 — Additional file 11. Supplementary figure legends. [file 13287_2023_3437_MOESM11_ESM.docx]

**Supplemental information**

**Title**

**Single-cell RNA sequencing of human non-hematopoietic bone marrow cells reveals a unique set of inter-species conserved biomarkers for native mesenchymal stromal cells**

**Authors**

Loïc Fiévet, Nicolas Espagnolle, Daniela Gerovska, David Bernard, Charlotte Syrykh, Camille Laurent, Pierre Layrolle, Julien De lima, Arthur Justo, Nicolas Reina, Louis Casteilla, Marcos J Araúzo-Bravo, Abderrahim Naji, Jean-Christophe Pagès, Frédéric Deschaseaux.

**Figure S1**

A: Overview of human bone marrow (BM) mapping processes as described in Methods (this figure was created by us). B: UMAP representation of human BM cell batch clusters identified via Azimuth analysis, showing the contribution of donor to each cluster. Note that both sexes (male and female) were considered. C: Correlation map of the whole clusters found by scRNA-seq. After calculations, the close relationship between each cluster was obtained. As expected, all erythroid populations (Eryth) were clustered as found for B-cell fractions and hematopoietic stem/progenitor cells (HSC/prog). D: UMAP of representative top genes of human clusters. The expression of genes typifying one cluster is depicted on the UMAP. Several hematopoietic subpopulations were detected despite the sorting protocol. This is not surprising because HSCs and some immature progenitors are CD45low. Some B-cell subpopulations can also express the CD45 at a low level according to their maturation stages. With the Park et al. studies on the determination of human hematopoietic cells at a single cell level ^1^ and in agreement with a recent study on single cells derived from a large panel of tissues in a human cell atlas (*Tabula sapiens*^2^), several clusters could be annotated as 1) multipotent (MP) myeloid progenitors with erythroid, granulo-monocyte and neutrophils potential (clusters 0, 1, 2, 3, and 7 or Eyth-prog, Myeloid Eryth-prog, HSC-Myeloid-prog, MP-Eryth-Granulo-prog, and MP-Eryth-Myeloid-prog, respectively), 2) MP-lymphoid progenitors (clusters 5 and 12 or Common Lymphoid-prog and lymphoid MP prog, respectively), 3) plasma cells (cluster 4), 4) HSC MP progenitors (HSC-MPP (cluster 9), 5) granulo-monocyte-neutrophile-restricted progenitors (cluster 8) and MP granulo-monocyte progenitors (cluster 6), and 6) lymphoid MP progenitors (cluster 12). Moreover, by using key markers of differentiation expressed in the erythroid lineage, we could easily identify erythroid progenitor clusters spanning a differentiation continuum with high expression of the mRNAs *CA1*, *HBB*, *GYPA* or *GATA1*. We identified two hemoglobin Delta chain-positive (*HBD*+) Eryth-prog and early Eryth-prog in clusters 0 and 1; *PPDPF*+ committed myeloid-eryth progenitors proliferating or not (in clusters 3 and 7); heterogeneous nuclear ribonucleoprotein H1 (*HNRNPH1+*) MP (MP) myeloid progenitors in cluster 2; and expression of high mobility group box 1 (*HMGB1*) marker encompassing all myeloid progenitors (clusters 0, 1, 2, 3, 6 and 7), a sign of more mature erythroid functionality. Of note, the highly immature populations 6, 8 and 9 typifying lysozyme (*LYZ*+) granulo-monocyte-neutrophyle progenitors (cluster 8), the whole defender against cell death 1 (*DAD1*+) myeloid progenitors (cluster 6) and signal recognition particle 72 (*SRP72+*) HSCs (cluster 9). In addition to HSCs and myeloid cells, we could discriminate other types of hematopoietic cells belonging to the lymphoid lineage such as B cells or more immature MP lymphocyte progenitors (MLPs) and committed common lymphocyte progenitors (CLPs). Different B-lymphoid cells were detected: memory B and plasma cells (clusters 4, 5) expressing *IGHG1* and *HLA-E* mRNAs and pre-B cells (cluster 12) expressing *VPREB1* and *CD79B* mRNAs. The analysis of these whole populations of selected cells highlighted several highly immature hematopoietic cell fractions mainly consisting of *HMGB1*+ myeloid or immunoglobulin kappa constant (*IGKC*+) lymphoid lineages.

**Figure S2.** For each subpopulation cluster, we calculated the genes with the highest expression after ranking them according to their significance as compared with the remaining population of detected cells (see Table).

**Figure S3.** Populations of cells constituting whole tissues used for scRNA-seq in the *T. sapiens* study^2^. This representation was obtained by using online tools provided by the group (https://tabula-sapiens-portal.ds.czbiohub.org/). We indicate by arrows the non-hematopoietic cell fractions that we detected in our study and how they were referred (i.e., endothelial cells [ECs], mural cells [MCs], and MSCs). ECs were found in endothelial clusters, MCs were referred to as smooth muscle cells (SMCs), pericytes and myofibroblasts; MSCs were defined as MSCs, fibroblasts or stromal cell clusters.

**Figure S4.** List of genes expressed by MSCs after comparison of our data and the Ye et al. data (**A**) ^3^ available by using their online tools in Single Cell Portal with the accession number SCP1747 (https://singlecell.broadinstitute.org/single_cell/study/SCP1747/bmn-characterization). (**B**) As depicted here, our MSC annotations were fitted with cluster 11. (**C**) With a Venn diagram, half of the genes we obtained were also found in this previous study.

**Figure S5, S6 and S7.** Protein–protein interaction (PPI) studies of our gene list typifying MSCs according to osteogenesis (Figure S5), adipogenesis (Figure S6) and hematopoiesis (Figure S7). PPI involved using Genomatix browser tools (<https://genomatix.de>). All genes detected by scRNA-seq expressed by the MSC fraction were found highly represented in osteogenesis, adipogenesis or hematopoiesis networks (yellow outlined).

**Figure S8, S9 and S10.** PPI of transcription factors (TFs) increased in expression in the different non-hematopoietic populations: ECs, MSCs, and MCs. Key TFs at the center of specific module are highlighted in green. These TFs were studied and discussed in the Results section. The PPI was obtained by uploading our main gene lists in the OmicsNet web browser (https://www.omicsnet.ca/).

**Figure S11.** Key TFs conserved between humans and mice calculated by PPI.

**Figure S12.** Immuno-staining of human bone marrow ECs from different samples of biopsies by anti-NHERF2 antibody.

**References**

1 Park, J. E. *et al.* A cell atlas of human thymic development defines T cell repertoire formation. *Science* **367**, doi:10.1126/science.aay3224 (2020).

2 Tabula Sapiens, C. *et al.* The Tabula Sapiens: A multiple-organ, single-cell transcriptomic atlas of humans. *Science* **376**, eabl4896, doi:10.1126/science.abl4896 (2022).

3 Ye, J. *et al.* Deconvolution of the hematopoietic stem cell microenvironment reveals a high degree of specialization and conservation. *iScience* **25**, 104225, doi:10.1016/j.isci.2022.104225 (2022).
